# Quantitative susceptibility mapping of the head-and-neck using SMURF fat-water imaging with chemical shift and relaxation rate corrections

**DOI:** 10.1002/mrm.29069

**Published:** 2021-11-30

**Authors:** Beata Bachrata, Siegfried Trattnig, Simon Daniel Robinson

**Affiliations:** 1 High Field MR Centre, Department of Biomedical Imaging and Image-Guided Therapy, Medical University of Vienna, Vienna, Austria; 2 Karl Landsteiner Institute for Clinical Molecular MR in Musculoskeletal Imaging, Vienna, Austria; 3 Centre of Advanced Imaging, University of Queensland, Brisbane, Australia; 4 Department of Neurology, Medical University of Graz, Graz, Austria

**Keywords:** chemical shift, fat-water, head-and-neck, QSM, SMURF

## Abstract

**Purpose:**

To address the challenges posed by fat-water chemical shift artifacts and relaxation rate discrepancies to quantitative susceptibility mapping (QSM) outside the brain, and to generate accurate susceptibility maps of the head-and-neck at 3 and 7 Tesla.

**Methods:**

Simultaneous Multiple Resonance Frequency (SMURF) imaging was extended to 7 Tesla and used to acquire head-and-neck gradient echo images at both 3 and 7 Tesla. Separated fat and water images were corrected for Type 1 (displacement) and Type 2 (phase discrepancy) chemical shift artefacts, and for the bias resulting from differences in T_1_ and ${\text{T}}_{2}^{*}$ relaxation rates, recombined and used as the basis for QSM. A novel phase signal-based masking approach was used to generate head-and-neck masks.

**Results:**

SMURF generated well-separated fat and water images of the head-and-neck. Corrections for chemical shift artefacts and relaxation rate differences removed overestimation of the susceptibility values, blurring in the susceptibility maps, and the disproportionate influence of fat in mixed voxels. The resulting susceptibility maps showed high correspondence between the paramagnetic areas and the locations of fatty tissues and the susceptibility estimates were similar to literature values. The proposed masking approach was shown to provide a simple means of generating head-and-neck masks.

**Conclusion:**

Corrections for Type 1 and Type 2 chemical shift artefacts and for fat-water relaxation rate differences, mainly in T_1_, were shown to be required for accurate susceptibility mapping of fatty-body regions. SMURF made it possible to apply these corrections and generate high-quality susceptibility maps of the entire head-and-neck at both 3 and 7 Tesla.

## 1 Introduction

Quantitative susceptibility mapping (QSM) is a method for calculating the distribution of the magnetic susceptibilities of tissue from a fieldmap (Δ*B*_0_)—the difference between the externally applied magnetic field and the demagnetisation field generated by the tissue itself.^1^ The phase of the gradient-echo (GRE) signal is used to generate the fieldmap, which is converted to a map of susceptibility by removal of background fields and dipole inversion. Generation of an accurate fieldmap is a crucial step in the process, as errors generally propagate throughout the QSM analysis pipeline and bias susceptibility estimates.^2^

QSM provides excellent contrast in the imaging of calcifications, hemorrhages, and iron depositions, as well as tissue microstructure and oxygenation.^3^ The technique has found application including the assessment of Alzheimer and Parkinson disease, Multiple Sclerosis (MS), the grading of tumors and monitoring of their growth,^4–6^ and the past decade has seen an increase of interest in QSM outside the brain: in the liver,^7–11^ breast,^3,12,13^ kidney,^14^ prostate,^15–17^ heart,^18–20^ knee,^21^ trabecular bone,^22^ spine,^23^ and the entire head-and-neck region.^24–29^ Compared to brain-only QSMs, susceptibility mapping of the head-and-neck allows the visualization of deep gray matter nuclei in the brainstem^30^—potential targets for deep brain stimulation^31,32^—MS lesions^33^ in the brainstem, and visualization and assessment of tumors in the facial skull; epi-, naso-, and oropharynx; the base of the tongue and mouth; salivary glands; and possible regional lymph nodes.^34^ The presence of fat in these regions, however, generally results in errors in field estimation that originate in several effects. First, because of the circa 3.4 ppm^35^ chemical shift difference between fat and water, the fat image is shifted relative to water along the frequency-encoding direction by a number of voxels given by

$$N_{voxels}=\frac{\Delta f}{rBW/pixel},$$

where *rBW/pixel* is the receiver bandwidth per pixel and Δ*f* the fat-water chemical shift. This so-called Type 1 (displacement) chemical shift artifact (CSA) leads, in addition to the errors in the spatial distribution of susceptibility sources, also to areas of signal voids, making spatial phase-unwrapping problematic. While QSM generally benefits from the use of higher field strengths, allowing either shorter acquisition times or higher resolution,^36^ the increased fat-water chemical shift difference leads to a more pronounced Type 1 CSA. Second, the different precession frequency of fat with respect to water gives rise to an echo time (TE) -dependent phase component (*φ*) in GRE, defined as:

Δφ(TE)=2πΔfTE,

which does not reflect the tissue susceptibility and also causes destructive interference between water and fat signals—the so-called Type 2 (phase discrepancy) CSA. Last, differences in T_1_ and ${\text{T}}_{2}^{*}$ relaxation times lead the acquired water and fat signals to be weighted differently, according to the signal equation:

$$S=\frac{PD(1-e^{-TR/T_{1}})e^{-TE/T_{2\text{Sin}\alpha }^{*}}}{1-e^{-TR/T_{1\text{COS}\alpha }}}$$

with *PD* being the proton density and *a* the flip angle (FA). The proton density ratio of the contributing water-based and fat-based tissues is biased by this signal weighting, which skews field estimates in mixed voxels.

To reduce the effects of Type 1 CSA, high receiver bandwidths are conventionally used, allowing only a partial correction and coming at the price of decreased SNR. To reduce Type 2 CSA, an acquisition at the in-phase TEs can be used,^12,23,24,26^ although this restricts the choices of TEs. The effects of relaxation time differences are known to bias the estimation of proton density fat-fraction (PDFF),^37,38^ but are generally neglected in QSM.^12,23,24,28^ To minimize the bias caused by T_1_ relaxation rate differences, small FAs have to be used, resulting in poor SNR, or a correction based on known T_1_ values^38^ can be applied. In the case of PDFF mapping of the iron overloaded liver, the differences in ${\text{T}}_{2}^{*}$ are assumed to be negligible^39^ due to the shortening of the values being dominated by the presence of iron. It remains to be established if this assumption holds in other cases or if a correction should be applied.

As an alternative to in-phase imaging, the Dixon approach^40^ can be used, allowing the simultaneous generation of water-only and fat-only images from a multi-echo acquisition. Unlike in-phase imaging, Dixon allows PDFF maps to be generated simultaneously and the multi-peak fat spectrum to be considered. The Dixon approach is, however, problematic in voxels with fat-fractions close to 50%,^23^ which may occur either in mixed tissues such as the parotid glands or, due to partial volume effects, at the edges of water-based and fat-based structures. The Dixon approach also requires a very short echo spacing to achieve robust fat-water separation,^41,42^ which is suboptimal for QSM, where the maximal contrast-to-noise ratio (CNR) is achieved at the TE close to the ${\text{T}}_{2}^{*}$ of the tissue.^43^ To overcome this problem and also the problem over-smoothing of the fieldmaps inherent to most of the Dixon approaches, two methods have been developed specifically for Dixon-based susceptibility mapping. The first method, simultaneous phase unwrapping and removal of chemical shift (SPURS),^9^ simultaneously unwraps the phase and removes chemical shift using single species fitting combined with graph cuts, while the second technique uses two trains of echoes with short and long echo spacings.^44^ However, none of the Dixon methods addresses the errors in susceptibility estimates resulting from Type 1 (displacement) CSA. In magnitude Dixon imaging, the separated water and fat images can be corrected for their relative displacement, allowing the generation of chemical shift displacement-free recombined fat-water images. The fieldmap, however, is generally generated as a by-product of the minimization step and, because Type 1 CSA affects the acquired multi-echo signal that is being fitted, it is present in the fieldmaps, to the detriment of QSM. The same is true for the in-phase imaging approach.

We have recently presented a Simultaneous Multiple Resonance Frequency (SMURF^45^) approach to fat-water imaging based on multi-band principles, which was applied to correct the CSAs in GRE and turbo spin-echo magnitude imaging at 3 T. In SMURF, multi-band pulses are applied together with CAIPIRINHA to allow separate images of fat and water to be generated. Prior to recombination, a complex summation of the fat and water signals, those signals can be corrected for Type 1 and Type 2 CSAs, and, if the relaxation times are known, for the bias resulting from fat-water relaxation rate differences. We hypothesized that the SMURF technique would yield similarly cleanly separated fat and water images at higher field strengths, as both field inhomogeneity and chemical shift difference generally scale with the field. Although a single-peak fat signal model is assumed in generating the spectrally selective radiofrequency (RF) pulses in SMURF, it has been shown that this approximation does not lead to significant bias in QSM outside the brain.^23^ Susceptibility values of fatty tissues vary, however, quite widely in the literature,^23^ e.g., 0.29 ppm^7^ and 0.57 ppm^9^ for the fat in the liver, 0.19 ppm^21^ for the fat in the knee, and 0.29 ppm^24^ for the fatty fascia in the head-and-neck. Nevertheless, in all cases was the fat assessed as being more paramagnetic than the surrounding water-based tissues.

In addition to the complications caused by the presence of fat, QSM outside the brain is also made problematic by the difficulties in generating the tissue masks required by most QSM approaches. Unlike for the brain, there are no standard segmentation tools, and simple magnitude thresholding generally includes some voxels with unreliable phase, giving rise to artifacts in the susceptibility maps, and potentially excludes extended areas of interest where coil sensitivities are low (e.g., in the neck at brainstem at a field strength of 7 T and above). Even these low signal areas often show high coherence of the phase signal, however. This, and the fact that magnitude-based masks include voxels with high magnitude signal but unreliable phase (e.g., veins) have led to the suggestion to generate masks using phase image.^46^

The aim of this study was to address the challenges of QSM outside the brain and, using simultaneous fat-water imaging with SMURF, to generate chemical shift and relaxation rate bias-free, high CNR susceptibility maps of the entire head-and-neck region. Since QSM generally benefits from the use of higher field, we extended SMURF to 7 T, although the need for different RF excitation pulses and the modified field variations this a non-trivial translation. Additionally, we aimed to develop a simple but robust approach for generating head-and-neck masks using the signal phase.

## 2 Methods

Measurements were performed with a 3T Siemens Prisma scanner (*syngo* MR VE11C, Siemens Healthineers, Erlangen, Germany) and a 7T Siemens MAGNETOM Plus scanner (*syngo* MR VE12U AP01). The head-and-neck region of 10 healthy volunteers was scanned—five volunteers at 3 T (V1–V5) using a 64-channel Siemens head-and-neck coil and five at 7 T (V6-V10) using a 32-channel head coil (Nova Medical, Wilmington, Massachusetts, USA). The 3T and 7T data were acquired to evaluate the performance of SMURF imaging at each field strength independently and to assess the effectiveness of the corrections for chemical shift and relaxation rate biases at the most commonly used field strengths in QSM, but not with the intention of comparing results between the two field strengths. Written informed consent was provided by all the participants, and the study was approved by the Ethics Committee of the Medical University of Vienna.

### 2.1 RF pulse design

Least-squares-filtered minimum-phase Shinnar-Le Roux pulses^47–49^ were designed using Vespa^50,51^ and used to create both bands of two dual-band pulses: one optimized for fat-water imaging at 3 T^45^ and one for 7 T.^29^ The larger chemical shift difference at 7 T allowed the use of broader and, hence, shorter minimum-phase SLR RF pulses with a duration of 8.0 ms compared to 11.8 ms at 3 T, allowing a first echo time of about 2.5–4.5 ms at 7 T compared to about 4.5–7.0 ms at 3 T.

### 2.2 Acquisition parameters

#### 2.2.1 3T measurements

Sagittal 3D multi-echo GRE SMURF images were acquired with anterior-posterior phase-encoding direction, field of view (FOV) = 230 × 230 mm, resolution = 1.3 × 1.3 × 1.3 mm, 128 slices, TE = {6.5, 13.0, 19.5, 26.0, 32.5} ms, repetition time (TR) = 45 ms, FA(water) = 15°, FA(fat) = 27°—the respective Ernst angles assuming T_1_ values of 400 ms and 1400 ms for fat and water respectively—rBW/pixel = 190 Hz and monopolar read-out. Parallel imaging acceleration of R = 3 and partial Fourier of 7/8 were applied, resulting in total acquisition time of 4 min 53 s.

#### 2.2.2 7T measurements

Sagittal 3D multi-echo GRE SMURF images were acquired with anterior-posterior phase-encoding direction, FOV = 230 × 230 mm, resolution = 1.0 × 1.0 × 1.0 mm, 160 slices, TE = {3.85, 7.70, 11.55, 15.40, 19.25, 23.10} ms, TR = 35 ms, FA(water) = 12°, FA(fat) = 20°—the respective Ernst angles assuming T_1_ values of 550 ms and 1700 ms for fat and water respectively—rBW/pixel = 360 Hz, and monopolar readout. Parallel imaging acceleration of R = 3 and partial Fourier of 6/8 were applied, resulting in total acquisition time of 5 min 13 s.

For all volunteers, a low-resolution conventional GRE scan (i.e., with broadband RF excitation) was acquired to calculate the GRAPPA kernel for fat-water separation using parallel imaging reconstruction. The imaging parameters of the prescans were: in-plane resolution = 3.6 × 3.6 mm, TE/TR = 1.5/3.5 ms at 3 T and TE/TR = 3.0/5.4 ms at 7 T, FA = 5°, rBW/pixel = 690 Hz at 3 T and rBW/pixel = 650 Hz at 7 T with the number of slices and slice thickness the same as in the main SMURF scans. To evaluate possible changes to image contrast, signal phase and resulting susceptibility maps from the use of SMURF RF pulses and processing, high-resolution conventional GRE scans, with the same imaging parameters as was used for the SMURF scans, were also acquired and susceptibility maps were calculated as described in the following section (Supporting Figures S1 and S2, which are available online).

### 2.3 Data analysis

**Figure 1:**
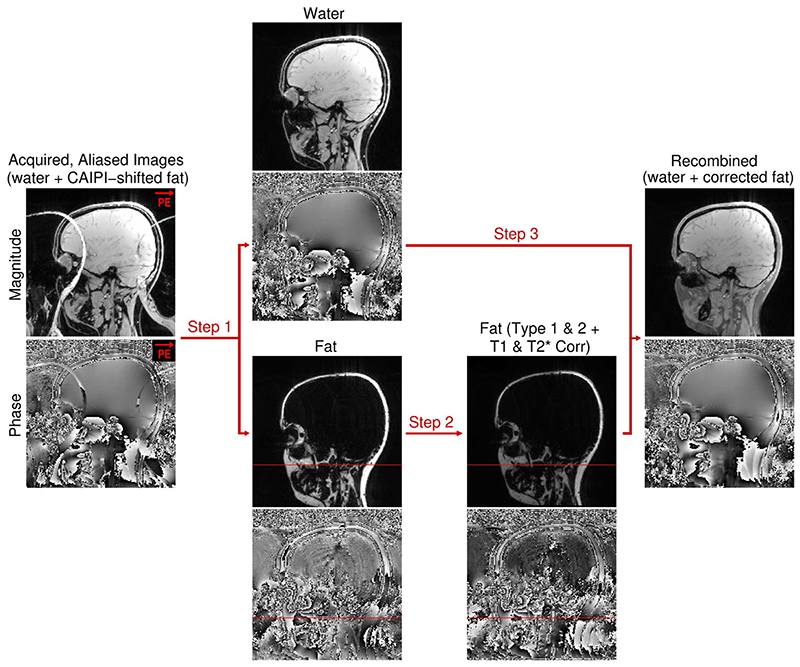
Image processing and postprocessing pipeline. In Step 1, overlapping water and CAIPIRINHA-shifted fat images are unaliased using slice-GRAPPA. In Step 2, the fat image is shifted to reverse chemical shift displacement (Type 1 chemical shift artefact)— see the position before and after the correction relative to the red reference line -, corrected for the chemical shift-related phase evolution (Type 2 chemical shift artefact) and rescaled to correct for the bias caused by T_1_ and ${\text{T}}_{2}^{*}$ relaxation rate differences. In Step 3, the complex fat and water images are recombined, generating a fat-water image free of chemical shift and relaxation rate discrepancies.

SMURF fat and water images were reconstructed from raw data in MATLAB (Mathworks Inc, Natick, MA) using slice-GRAPPA^52^ and coil-combination with ASPIRE.^53^ The fat complex images were corrected for Type 1 (displacement) and Type 2 (phase discrepancy) CSAs and for differences in T_1_ and ${\text{T}}_{2}^{*}$ relaxation rates (Figure 1). Literature values were used for the T_1_ correction (400/1400 ms; and 550/1700 ms for fat/water at 3 and 7 Tesla, respectively).^54–56^ For ${\text{T}}_{2}^{*}$ correction, separate ${\text{T}}_{2}^{*}$ maps of water and fat were generated and the ratio of fat and water signal weighting, in each voxel, was calculated. To avoid transferring noise from this ratio map to the images in a voxel-by-voxel correction, a single correction factor (CF), calculated as the median of the per-voxel ratios of fat and water signal weighting was used. The median was assessed only over voxels for which the fat fraction (FF) was in the range 0.1 ≤ FF < 0.8:

$$\text{CF}=\text{median}\left(\frac{e^{-TE_{iEcho}/{\text{T}}_{2fat}^{*}(r)}}{e^{-TE_{iEcho}/{\text{T}}_{2water}^{*}(r)}},\text{for}\;\text{all}\;r,\text{where}\;0.1<=FF(r)<0.8\right),$$

and the resulting CF was used for the correction of all mixed voxels.

**Figure 2:**
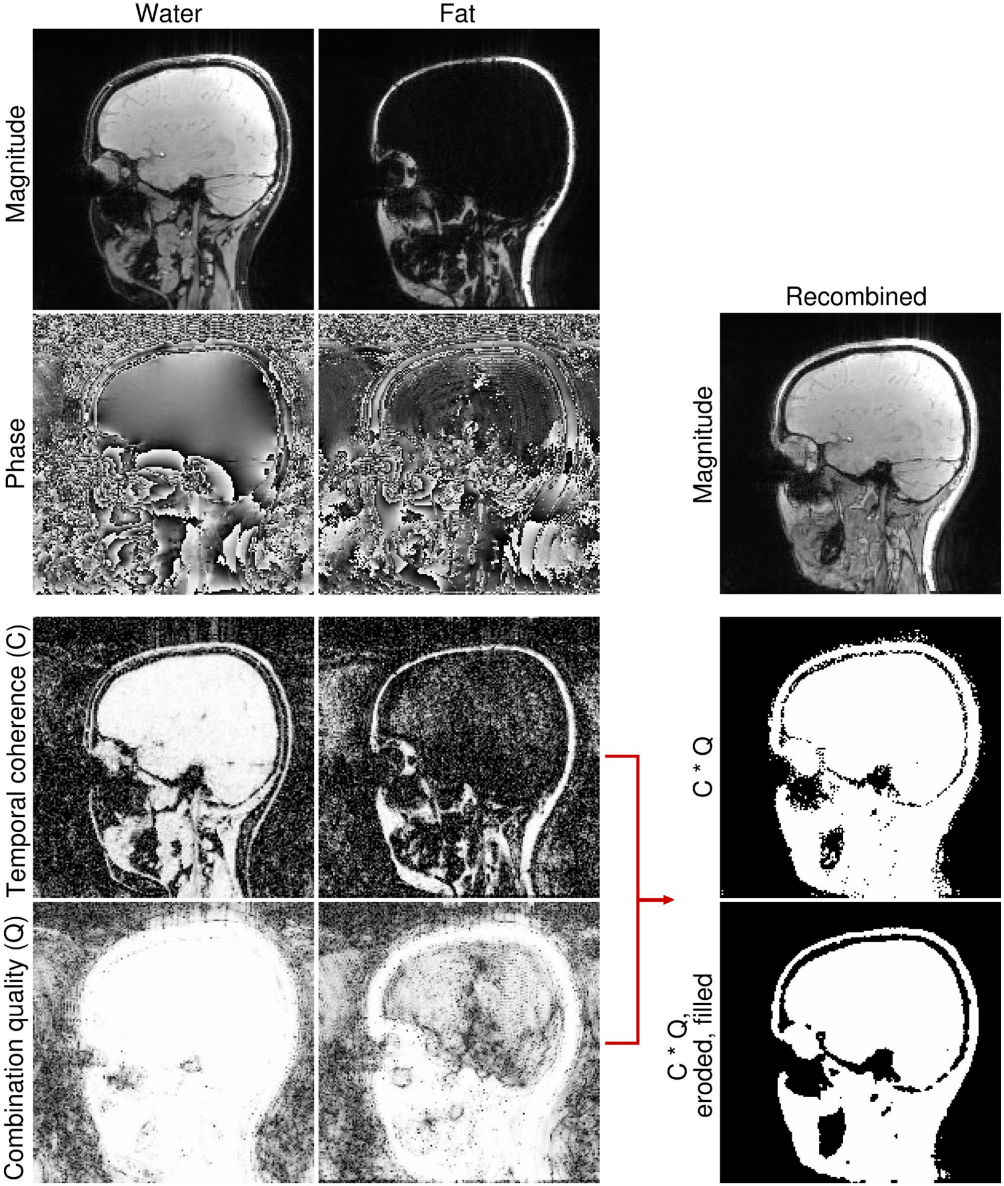
The generation of a head-and-neck mask using signal phase. Maps of temporal phase coherence (C) and phase combination quality (Q) are calculated, for fat and water separately, thresholded and combined to generate a single mask (C * Q). This mask is smoothed and thresholded to fill in small holes, eroded, and filled again. Note that the phase combination quality of the second echo is used to exclude voxels with very short relaxation times.

To generate a head-and-neck mask, maps of temporal phase gradient coherence (Weight 2 in Ref [57,58]) and phase combination quality, i.e., Q-metric,^59^ were calculated separately for fat and water. The temporal phase gradient coherence weight, calculated as:

$$C(i,j)=\max\left(0,1-\left|\Omega \left(\varphi_{i,TE(1)}-\varphi_{j,TE(1)}\right)-\Omega \left(\varphi_{i,TE(2)}-\varphi_{j,TE(2)}\right)\cdot TE(2)/TE(1)\right|\right),$$

with *i* and *j* representing two adjacent spatial locations and Ω the wrapping operator, describes how closely the time evolution of phase is to being linear, and the Q-metric, defined as:

$$Q=100\times \frac{\left|\sum_{M_{c}}M_{c}e^{i\varphi_{c}}\right|}{\sum_{c}M_{c}},$$

with *M_c_* representing the signal acquired by channel *c* of the receiver array, describes how well the phase offset-corrected signal is matched between the individual receiver channels. Since both of these metrics are low in the voxels with unreliable phase, these voxels tend to be excluded from masks. To exclude voxels with very short relaxation times, the Q-metric of the second echo was used. The fat and water maps of temporal phase gradient coherence (C) were summed to generate a joint fat-water quality map, which was thresholded to exclude voxels with unreliable phase (threshold value of 0.6). The fat and water combination quality (Q) maps were also thresholded (threshold value of 0.98) and combined using an “OR” operation. A joint fat-water mask was generated by combining the maps of temporal phase gradient coherence (C) and the thresholded quality maps (Q) using an “AND” operation (Figure 2). This mask was smoothed (using smoothn in Matlab^60,61^) and thresholded, to fill in small holes and remove small disconnected groups of voxels for example, in the subcranial region (threshold value of 0.6), eroded and filled again (smoothn with subsequent thresholding at 0.3). All threshold values, as well as the combination and order of individual processing steps, were arrived at empirically on the first datasets and used for all volunteers.

To generate susceptibility maps, the multi-echo images were combined over echoes using inverse-variance weighting.^29,62,63^ Phase unwrapping was performed using ROMEO.^57,58^ Background-field correction was performed with PDF^64^ and susceptibility calculation with STAR^65^ using the Sepia toolbox.^66^

To assess the need for multi-echo acquisitions, including longer TEs, in QSM imaging of the head-and-neck, QSMs generated from all echoes combined and QSMs generated from individual echoes were compared both qualitatively—by visual assessment of the image contrast, noise, and presence of artifacts—and quantitatively—via calculation of CNR within ROIs—given as:

$$\text{CNR}=\frac{\left|\mathrm{mean}\left({\text{ROI}}_{\text{sig }}\right)-\mathrm{mean}\left({\text{ROI}}_{\text{bkg }}\right)\right|}{\mathrm{stdev}\left({\text{ROI}}_{\text{noise }}\right)}.$$

Within the brain, the CNR was assessed over eight ROIs located in deep gray matter structures (ROI_sig_) relative to the neighboring tissue (ROI_bkg_) and within the neck, over eight ROIs located in fatty structures (ROI_sig_) relative to the neighboring water-based tissues (ROI_bkg_). In both cases, the noise was estimated as the standard deviation of the signal within an ROI located in a homogeneous area of white matter (ROI_noise_).

To evaluate the effects of the corrections for CSAs and relaxation rate biases, the susceptibility maps generated from SMURF images with and without the individual corrections were compared both qualitatively—by visual assessment of the correspondence between the location of fatty tissues in the magnitude images and the paramagnetic areas in the susceptibility maps—and quantitatively—via the standard deviations within 6 ROIs located in areas of homogeneous tissue (i.e., lymph nodes, neck muscles and parotid glands). Additionally, susceptibility values of the fatty tissue were assessed within one extensive ROI, drawn over several distant fatty-neck tissue areas, and compared over the corrections. The quantitative ROI analysis (assessment of CNR, the variance of the susceptibility estimates, and assessment of the susceptibility of fatty tissues) was performed for three 3T volunteers (V1–V3) and three 7T volunteers (V6–V8), and the mean values over volunteers were calculated and compared over the corrections.

## 3 Results

There was a very high degree of correspondence between the recombined SMURF fat-water images acquired at the “random” TEs and the conventional images acquired at the same “random” TEs; and between the “random” recombined SMURF fat-water images corrected for the Type 2 CSA and the conventional images acquired at the “in-phase” TEs. This demonstrates that the use of SMURF RF pulses and image reconstruction has no discernible effect on image contrast or signal phase and that the Type 2 CSA correction effectively removed the phase discrepancy between water and fat (Supporting Figures S1 and S2).

The proposed phase-based masking approach, based on the joint fat-water temporal coherence and phase combination quality (Q-metric^59^) effectively excluded noisy phase voxels but retained the voxels in the neck (Figure 2), where, due to decreased coil sensitivities, the magnitude of the signal was lower (mainly at 7 T, because the transmit efficiency of the 32-channel head coil is low in inferior regions, and receive coil coverage of the neck area is limited).

**Figure 3:**
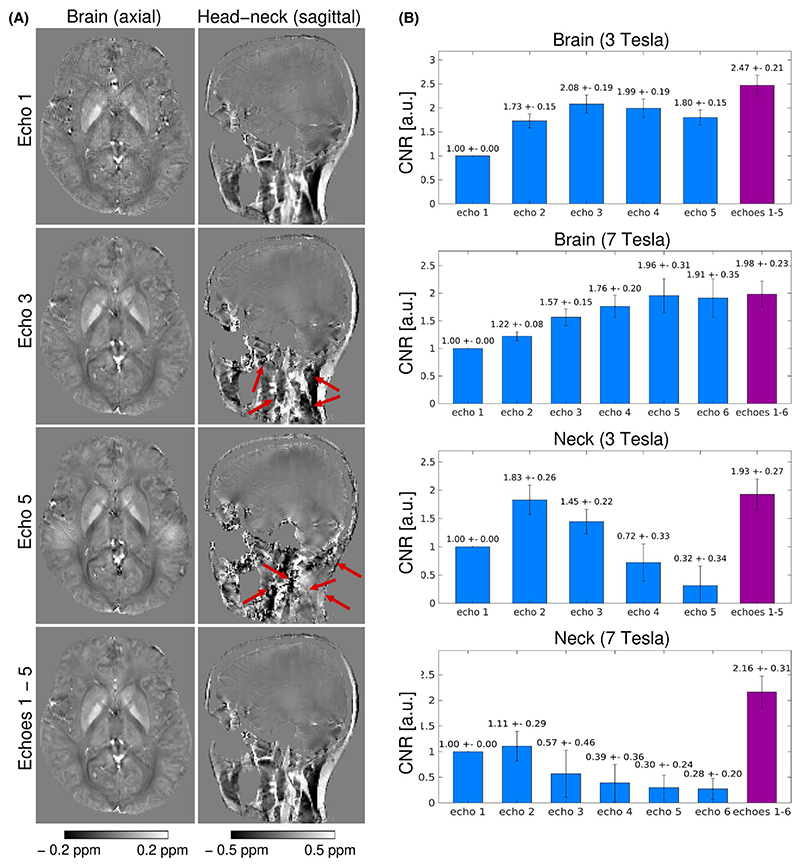
Comparison of single-echo and multi-echo susceptibility maps (A) and CNR quantification within the brain and the neck (B). A) 3T QSM images of brain-only (left column) and entire head-and-neck (right column) generated from the first (first row), third (second row), and fifth (third row) echo of the multi-echo acquisition, or from all echoes (bottom row) of the multi-echo acquisition of one 3T volunteer (V1). Note that, while the contrast of the susceptibility maps within the brain visibly increases and the image noise decreases with increasing TE, errors occur for later echoes outside the brain (red arrows). B) Quantification of the CNR within the brain (deep gray matter regions) and within the neck (fatty-neck regions) for the susceptibility maps generated from one respective echo of the multi-echo acquisition. The mean values and standard deviations of the relative CNR values over ROIs are normalized to the CNR of first-echo susceptibility maps and the mean values over the three volunteers (V1–V3 at 3 T and V6–V8 at 7 T) are displayed. While within the brain, the later echoes show higher CNR, in the neck higher CNR is achieved at the earlier echoes. The highest CNR is generally achieved when using all echoes combined.

The susceptibility maps generated from the first echo provided good tissue contrast in the areas of short ${\text{T}}_{2}^{*}$ , such as the neck and around sinuses, but in the brain, the contrast was low and the maps were noisy (Figure 3A, top row). On the other hand, the susceptibility maps generated from the later echoes showed improved contrast and decreased noise in the brain, but artifacts were present in the neck (Figure 3A, second and third rows). The QSMs generated from all echoes combined showed high contrast and low noise over the entire head-and-neck region (Figure 3A, bottom row). This was confirmed by the quantitative analysis; within the brain, the CNR was higher at the later echoes (Figure 3B, top two rows), but within the neck, the later echoes showed very low CNR and high variance, suggesting unreliability of the results (Figure 3B, bottom two rows). Using all echoes combined yielded high CNR in all cases. Within the brain ROIs, the CNR peaked, for the 3T acquisition, between the third and fourth echo (TEs of 19.5 ms and 26.0 ms), and for the 7T acquisition, between the fifth and sixth echo (TEs of 19.3 ms and 23.1 ms).

**Figure 4:**
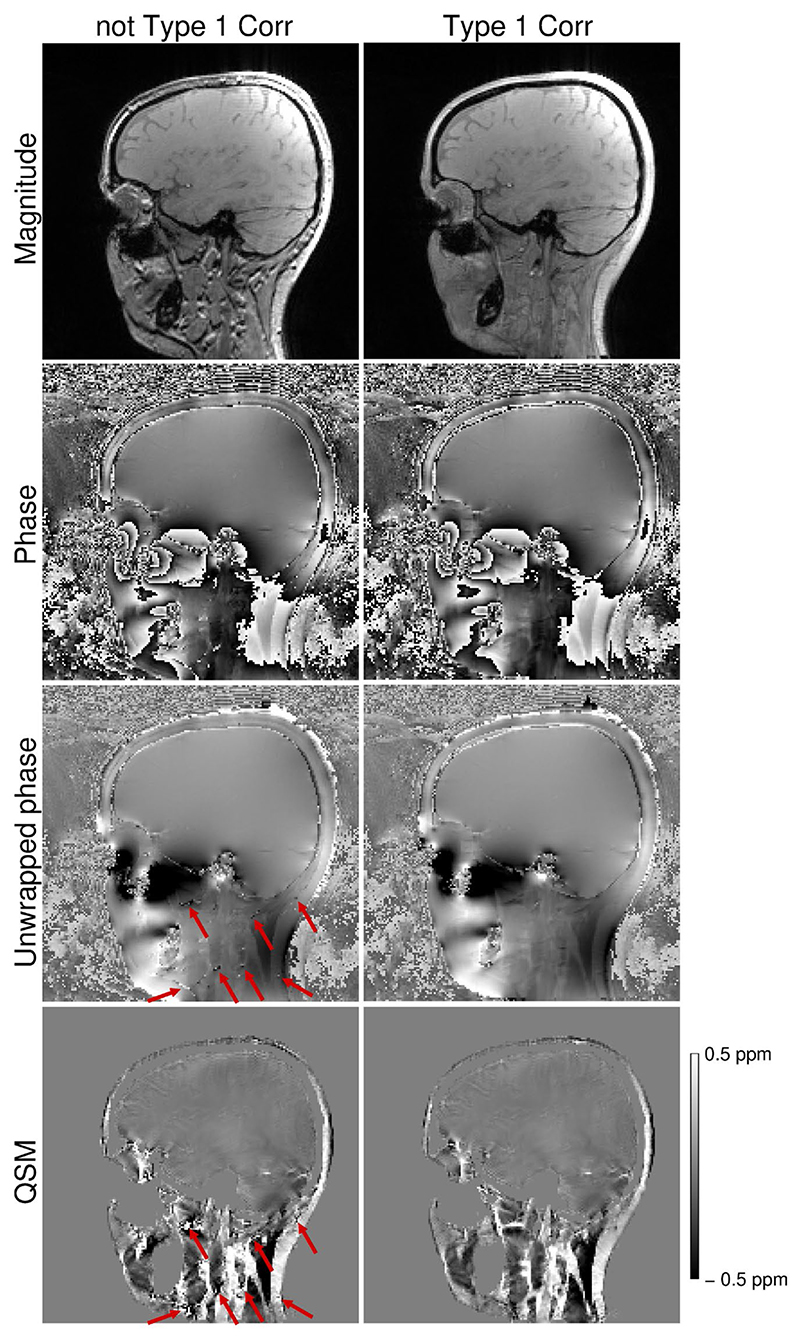
Comparison of phase unwrapping performance when applied to the images recombined without (left column) and with (right column) the correction for the Type 1 (displacement) chemical shift artefact. Note the erroneously unwrapped voxels (third row, red arrows) in the areas, where the displacement of fat results in areas of signal voids (first row) with unreliably defined phase (second row), which lead to artifacts in the susceptibility map (bottom row, red arrows).

If no Type 1 (displacement) CSA correction was applied, phase unwrapping errors occurred (Figure 4, third row) in areas where the displacement of fat led to signal voids with unreliable phase (Figure 4, second row), causing artifacts in the susceptibility maps (Figure 4, bottom row).

The SMURF method achieved generally well-separated fat and water images of the head-and-neck region at both 3 and 7 Tesla. For some volunteers, the larger field inhomogeneity at 7 T resulted in suboptimal fat-water separation in the very inferior or caudal area of the neck, manifesting as a local loss of water signal and swap of fat signal to the water image or the other way around (depending on the sign of the local field deviation) (Supporting Figure S3). These were generally easy to identify and located outside the area of interest, however.

**Figure 5:**
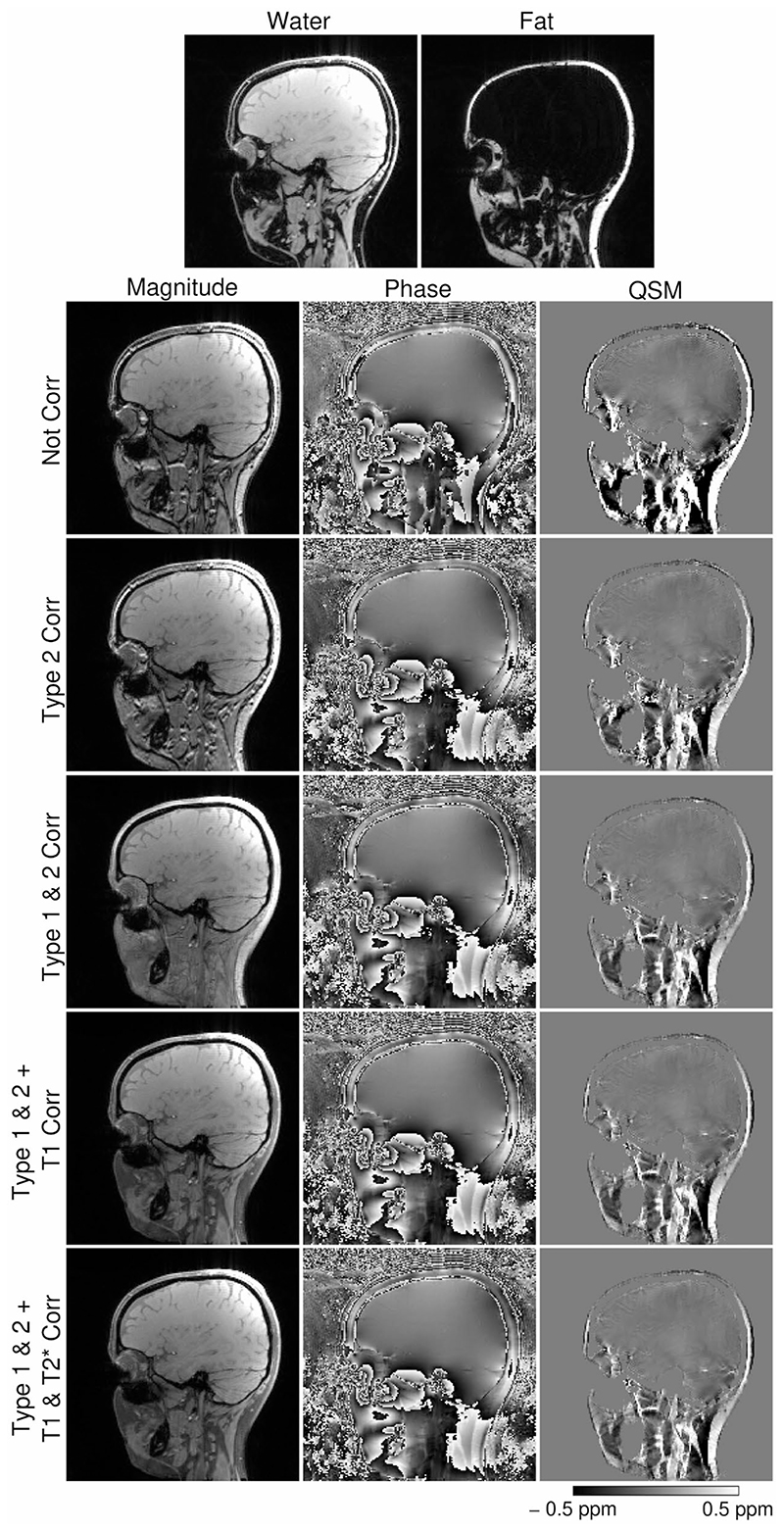
Illustration of the effects of corrections for chemical shift artefacts and for bias due to fat-water relaxation rate discrepancy, shown on the magnitude, phase and QSM images of one 3T volunteer (V1). The susceptibility maps generated from the images not corrected for chemical shift effects (row 1) are blurred, and the values are much higher than when corrected for the Type 2 CSA (row 2). The susceptibility maps corrected for the Type 1 and Type 2 CSAs clearly depict the paramagnetic fatty areas (row 3). Corrections for the fat-water differences in T_1_ (row 4) and ${\text{T}}_{2}^{*}$ (row 5) relaxation rates remove the erroneous domination of fat signal in the mixed voxels. Note the small effect of the ${\text{T}}_{2}^{*}$ correction.

Figure 5 illustrates the fat-water separation quality achieved by SMURF and the effects of the individual corrections for CSAs and relaxation rate biases on the magnitude, phase, and QSM. Without Type 2 (phase discrepancy) CSA correction (Figure 5, first column), the phase discrepancy between fat and water led to signal cancellation in mixed voxels—’India ink’ artifact outlining the fat-water borders. Additionally, the susceptibility values in fatty tissues were higher than with the correction applied. Without the Type 1 (displacement) CSA correction (Figure 5, second column), magnitude images contained areas of fat-water signal overlap as well as signal voids, and susceptibility maps at and around the fat-water borders were blurred and contained artifacts resulting from phase unwrapping errors. These were removed by the application of Type 1 CSA correction (Figure 5, center column). Corrections for fat-water relaxation rate bias (Figure 5, right two columns), primarily in T_1_, removed the fat hyperintensity (the fact that the fat signal was dis-proportionately large compared to the relative proton densities of the fat and water) and, thus, excessive influence on mixed voxels. This resulted in susceptibility maps with a high correspondence between the strongly paramagnetic areas and the locations of fatty tissue structures.

**Figure 6:**
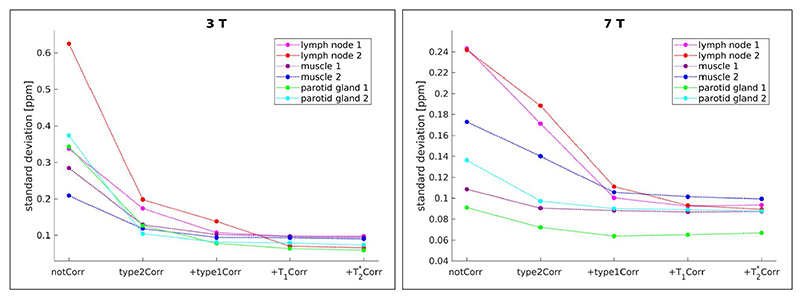
Standard deviation of QSM values within ROIs located in areas of homogeneous tissue; mean values over three subjects (V1-V3 and V6-V8 at 3 and 7 Tesla, respectively) are shown. In all ROIs, both at 3 T (left) and 7 T (right), the SD of QSM values is highest when no corrections for the CSAs and relaxation rate biases are applied and decreases with each correction. Note the largest effects attributable to CSA corrections, followed by correction of T_1_ bias.

**Figure 7:**
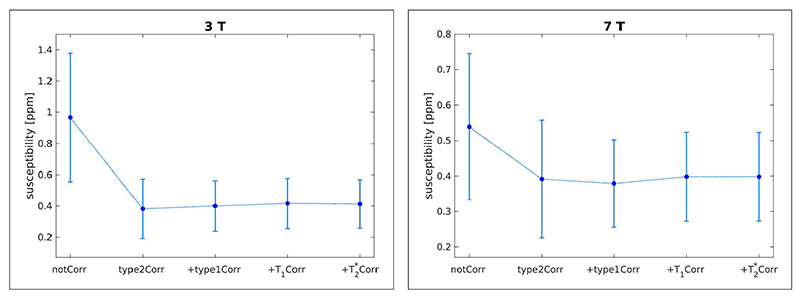
Susceptibility estimates of fatty tissue in the neck at 3 T (left) and 7 T (right); mean values over three subjects (V1-V3 and V6-V8 at 3 and 7 Tesla, respectively) are shown. Without correction for the Type 2 (phase discrepancy) chemical shift artefact, the susceptibility estimates are much higher than with the correction, median susceptibilities of 0.97 ppm and 0.54 ppm without corrections versus 0.41 ppm and 0.40 ppm with all corrections applied, at 3 T and 7 T respectively. The variance of the estimates over the ROI is also much higher without the Type 2 CSA correction.

The quantitative analysis also showed that the standard deviation of the QSM values within ROIs, which were located in areas of homogeneous tissue, was reduced when the corrections were applied (Figure 6), with the corrections for Type 1 and Type 2 CSAs having the greatest effects. Without the Type 2 (phase discrepancy) CSA correction, the susceptibility estimates of fatty tissue in the neck were significantly higher than with the correction applied (Figure 7): mean values over the three subjects of 0.97 ppm and 0.54 ppm versus 0.38 ppm and 0.39 ppm at 3 and 7 Tesla, respectively. With all corrections, the susceptibility estimates of fatty neck tissue were 0.41 ppm and 0.40 ppm at 3 and 7 Tesla, respectively.

**Figure 8:**
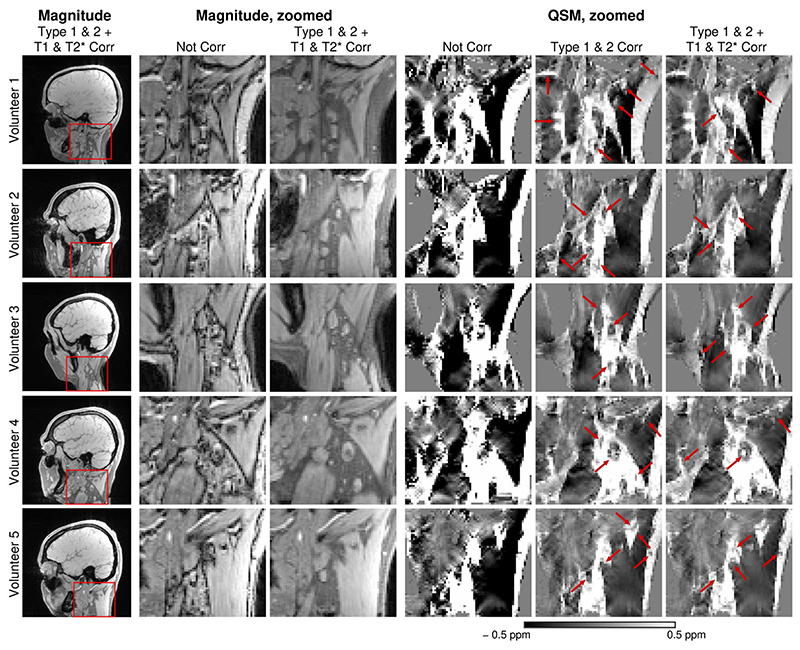
Susceptibility maps of all 3T volunteers (V1–V5) generated without any correction and with the corrections for chemical shift artefacts and bias due to fat-water relaxation rate discrepancies applied. Without the corrections (column 4), the susceptibility maps are blurred and the values are, compared to literature values, overestimated. The susceptibility maps corrected for the Type 1 and Type 2 CSA clearly depict the paramagnetic fatty areas (column 5, red arrows). Corrections for the fat-water differences in T_1_ and ${\text{T}}_{2}^{*}$ relaxation rates remove the dominant influence of fat in the mixed voxels (column 6, red arrows).

**Figure 9:**
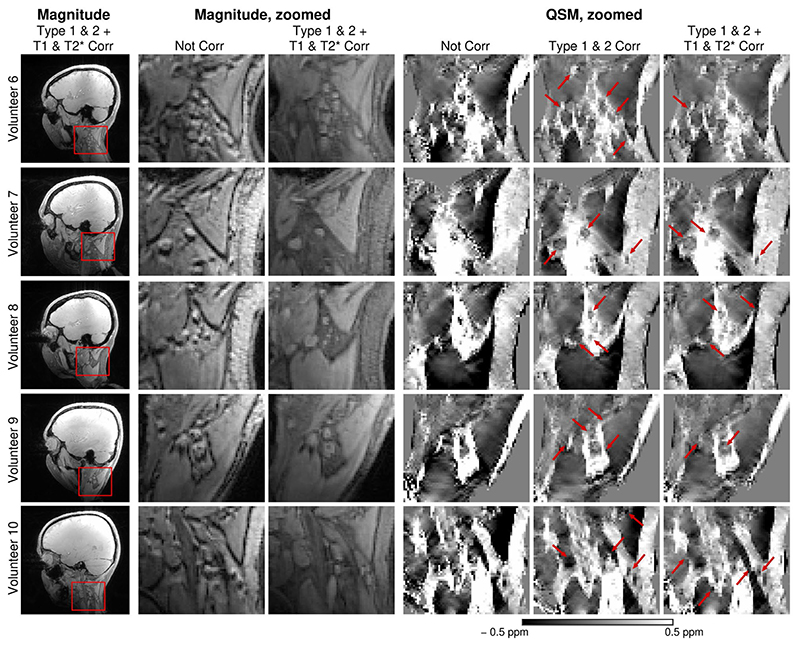
Susceptibility maps of all 7T volunteers (V6-V10) generated without any correction and with the corrections for chemical shift artefacts and bias due to fat-water relaxation rate discrepancies applied. Without the corrections (column 4), the susceptibility maps are blurred and the values are, compared to literature values, overestimated. The susceptibility maps corrected for the Type 1 and Type 2 CSA clearly depict the paramagnetic fatty areas (column 5, red arrows). Corrections for the fat-water differences in T_1_ and ${\text{T}}_{2}^{*}$ relaxation rates remove the dominant influence of fat in the mixed voxels (column 6, red arrows).

The estimated ${\text{T}}_{2}^{*}$ values of fat and water in mixed voxels were similar (Table 1), resulting in voxel-specific ratios of the signal weightings close to 1. Consequently, the ${\text{T}}_{2}^{*}$ bias correction, in which a correction factor, calculated as a median of these ratios over all mixed voxels was used, had only a modest effect (Figures 5, 8, and 9).

Figures 8,9 and Supporting Figures S4–S13 illustrate the effects of the corrections on susceptibility values for all 3T and 7T volunteers, respectively, and demonstrate that chemical shift artefacts and the bias due to fat-water relaxation differences have to be eliminated to generate susceptibility maps without gross artifacts. Without the Type 2 (phase discrepancy) CSA correction (third columns), the susceptibility values of fatty tissues were visibly higher and without the Type 1 (displacement) CSA correction (fourth columns), the susceptibility maps were more blurred around the fat-water borders and contained artifacts resulting from phase unwrapping errors. Corrections for fat-water relaxation rate bias (last two columns), primarily in T_1_, removed the excessive influence of fat signal on mixed voxels. With all corrections applied, the susceptibility maps showed a high degree of correspondence between the locations of paramagnetic areas and fatty structures. Note that the application of the corrections significantly improved the quality of the susceptibility maps even in the volunteers with only a small amount of fat in the neck.

## 4 Discussion

The challenges of quantitative susceptibility mapping outside of the brain, in regions that typically contain significant amounts of fat, have been addressed using a recently developed Simultaneous Multiple Resonance Frequency (SMURF) imaging approach combined with corrections for chemical shift and relaxation effects and a novel masking method. This approach led to high-quality susceptibility maps of the entire head-and-neck region, both at 3 and 7 Tesla, with a strong correspondence to the underlying anatomy.

In this study, we adapted the SMURF approach for 7 T, where due to the larger chemical shift difference, shorter RF pulses of 8 ms compared to roughly 12 ms at 3 T, could be used. This allowed a reduction in the minimum TE, roughly 2.5–4.5 ms instead of 4.5–7.0 ms at 3 T (depending on the receiver bandwidth and resolution), and a reduction in echo spacing. The faster-decaying signal at higher field strength could, thus, be captured and high-quality susceptibility maps of the head-and-neck could be generated both at 3 and 7 Tesla. The CNR of deep gray matter structures peaked, for the 3T measurements, between the third (TE = 19.5 ms) and fourth (TE = 26.0 ms) echo of the five echoes acquired and, for the 7T measurements, between the fifth (TE = 19.3 ms) and sixth (TE = 23.1 ms) echo of the six echoes acquired. This demonstrates that the later echoes are required to achieve high CNR in the brain. On the other hand, in the neck the CNR peaked at the earlier echoes, the second echo at both 3 T (TE = 13.0 ms) and 7 T (TE = 7.7 ms). This shows that, to achieve susceptibility maps with high CNR in the cerebrum, cerebellum, brainstem, as well as in the non-brain regions of the neck, both earlier and later echoes have to be acquired and combined with an effective echo combination approach, such as inverse variance weighting.^29,62,63^

A similar fat-water separation quality was expected to be achieved at 3 and 7 Tesla as both the field inhomogeneity and the chemical shift scale with field strength. In fact, while at 3 T correct fat-water separation was achieved in all cases over the entire head-and-neck (in addition to the subcutaneous region of the caudal neck area of two volunteers), at 7 T, some fat-water swaps occurred in the very inferior or caudal area of the neck for all volunteers. The head-and-neck represents, however, a challenging region in which the numerous interfaces between bones, air in sinuses, ears, and pharynx and soft tissue create strong susceptibility artifacts, leading to difficulties in shimming. In our previous study, well-separated fat and water images of breast, knee, and abdomen could be generated at 3 T.^45^ These results for the head-and-neck suggest that advanced shimming methods may be needed to be applied to 7T SMURF imaging of problematic body regions to exploit the benefits of the inherently higher SNR and shorter required echo trains.

We have shown that the removal of chemical shift artefacts and T_1_ relaxation differences is crucial to generating susceptibility maps without gross artifacts, and have demonstrated the sizeable effects of Type 2 (phase discrepancy) CSA and of the often neglected corrections for Type 1 (displacement) CSA and for bias due to fat-water T_1_ differences. Despite the absence of a ground truth against which to compare susceptibility estimates, a reduction in the variance over ostensibly homogeneous tissues and improved agreement with literature values points to the effectiveness of the corrections and the veracity of the results. Without the corrections, the susceptibility maps were blurred at fat-water borders and the values were, compared to those in the literature (0.2–0.6 ppm), overestimated (medians of 0.97 ppm and 0.54 ppm for 3 and 7 Tesla, respectively). With the corrections applied, these artifacts were removed (medians of 0.41 ppm and 0.40 ppm for 3 and 7 Tesla, respectively) and a high degree of correspondence was achieved between the location of fatty tissues and the strongly paramagnetic areas of susceptibility maps. Additionally, the standard deviation of susceptibility values across the ROIs located in areas of homogeneous tissue was reduced, suggesting lower levels of noise and artifacts. Although in-phase imaging^12,23,24,26^ or the Dixon approach^9,13,25,44^ can be used to eliminate Type 2 (phase discrepancy) CSA in QSM, Type 1 (displacement) CSA and T_1_ relaxation bias cannot be addressed with those approaches, as explained in the introduction. Chemical shift and relaxation rate effects can be reduced, but not eliminated, using high receiver bandwidths and low flip angles, but this comes at the price of decreased SNR. With SMURF, Type 1 and Type 2 chemical shift artefacts and T_1_ relaxation bias could be corrected and artifact-free susceptibility maps of the head-and-neck could be generated.

In this work, the T_1_ bias correction was performed using literature values of fat and water and led to QSMs with better correspondence to the underlying anatomy. Alternatively, and more precisely, T_1_ mapping could be carried out and measured T_1_ values could be used for correction. This would eliminate the need for the assumption that T_1_ values are the same for all water-based and all fat-based tissues present in the mixed voxels, but would require T_1_ mapping to be performed for both fat and water.

The ${\text{T}}_{2}^{*}$ values of fat and water in mixed voxels were shown to be quite similar, leading to correction factors for the disparate ${\text{T}}_{2}^{*}$ weighting of fat and water signals, which were close to 1. The similarity in fat-water ${\text{T}}_{2}^{*}$ values suggests, given the known differences in their T_2_ (48 ms and 29 ms, and 46 ms and 23 ms for fat and water at 3 and 7 Tesla, respectively),^55^ that the field inhomogeneity contribution dominates over the T_2_ contribution to ${\text{T}}_{2}^{*}$ . This concurs with observed ${\text{T}}_{2}^{*}$ estimates in mixed voxels (at 3 T, of 16 ms and 14 ms for fat and water respectively, and at 7 T, of 9 ms for both) being much lower than the literature T_2_ values. Although this disparity is generally increased for later echoes, later echoes contributed little to the combined phase, and thereby also to the QSMs, because of short ${\text{T}}_{2}^{*}$ constants and inverse-variance weighting. The effect of the ${\text{T}}_{2}^{*}$ correction on susceptibility maps was correspondingly small.

The proposed masking approach, which is based on the signal phase, specifically, the product of temporal phase coherence and phase combination quality (Q-metric), was shown to provide a simple means of generating a head-and-neck mask. A similar phase-based approach has been adopted by Stewart et al,^46^ who used thresholded spatial phase coherence. In the presence of fat, however, the spatial coherence would be low at the fat-water borders due to the fat-water phase discrepancy resulting from their susceptibility differences. Although this approach could be applied to the fat and water images separately, the values would be low at tissue edges. In both cases, using spatial phase coherence would, thus, lead to the exclusion of voxels at the fat-water border. Similarly, if the temporal phase coherence were to be calculated directly for the combined fat-water image, the resulting temporal phase coherence maps would be low at the fat-water borders, because of the fat-water susceptibility difference leading to a different temporal evolution of fat and water phase signal. The border voxels would be, thus, excluded from the mask even if Type 2 CSA correction or in-phase acquisition would be used (Supporting Figure S14). To avoid excluding these voxels from the mask, the temporal phase coherence maps of the separated fat and water signals had to be calculated and then combined. The proposed approach was shown to generate a mask that included most of the anatomy of interest and excluded noisy voxels. Nevertheless, if masks were to be needed in more problematic areas still, such as the hearing nerve in a study of acoustic neuroma, for instance, SMURF could be combined with a total-field in-version (TFI) QSM approach.^25,67^

As demonstrated in this study, SMURF opens up an alternative approach to QSM outside the brain. In addition to the head-and-neck region, SMURF could be also used for chemical shift and relaxation bias-free susceptibility mapping of the liver, kidney, joints, breast, spine, and other organs and regions. Although in-phase imaging^12,23,24,26^ and Dixon imaging^9,13,25,44^ are often used in these cases to remove Type 2 (phase discrepancy) CSA, we have shown that the correction of Type 1 (displacement) CSA and also of the bias due to fat-water relaxation differences, which cannot be performed with those approaches, is required for accurate susceptibility mapping. Additionally, to reduce shading artifacts, separate susceptibility maps for fat and water could be calculated separately from SMURF images and then combined.^17^ This would, however, make the approach more sensitive to masking and would benefit from, and likely be dependent upon, a TFI approach. Recently, a new preconditioned water-fat TFI (wfTFI) algorithm^68^ was developed and used to reduce background field-removal artifacts, noise amplification, and streaking artifacts in QSM of water-fat regions. A graph-cut^25^ (Dixon style) approach was used to separate water and fat and to generate a fieldmap and an R_2_^*^ map, which were then used as the basis for the QSM inversion. Potentially, SMURF could be also combined with wfTFI, which would also allow Type 2 CSA and relaxation bias to be eliminated.

In addition to GRE-based QSM of non-brain regions, SMURF would also bring benefits to QSM of the brain performed with EPI-based sequences. EPI and 3D EPI, in particular, are attracting increasing interest in QSM due to acquisition speed and the possibility to simultaneously map structure and function.^69–74^ In EPI, however, the low receiver bandwidth along the phase-encoding direction results in a large chemical shift displacement of the fat signal, which can shift fat from the skull into the brain and subcutaneous fat in the neck such that it overlays inferior brain regions such as the brainstem. Fat-suppression could be used, although the high specific absorption rate (SAR) demand of fat-sat pulses at ultrahigh field (UHF) make fat-water imaging an appealing solution^75^ and one that would retain fat information for QSM of non-brain regions and also possibly allow the use of fat images as navigators for prospective motion correction^76^ and dynamic B_0_ shimming.^77^

We have shown that CSAs and relaxation rate biases, that adversely affect the reliability of QSM in fatty regions, can be reduced using fat-water imaging with SMURF. This approach was shown to generate high-quality susceptibility maps of the head-and-neck at 3 and 7 Tesla with a strong correspondence to the known anatomy.

## 5 Conclusions

The challenges of QSM outside of the brain, in regions that typically contain significant amounts of fat, have been addressed with SMURF fat-water imaging with corrections for Type 1 and Type 2 CSAs and for relaxation rate biases. Unlike conventional imaging with broadband excitation, this approach was shown to be required to generate susceptibility maps of fatty regions without gross artifacts and with high correspondence between the paramagnetic areas and the locations of fatty tissues.

## Supplementary Material

Supplementary Information

## Figures and Tables

**Table 1:** Fat and water ${\text{T}}_{2}^{*}$ values and the weighting bias correction factor. Median values over all mixed voxels with fat fractions in the range 0.1 ≤ FF < 0.8 are shown. Note the similarity of ${\text{T}}_{2}^{*}$ values of fat and water, resulting in the weighting bias correction factors being close to 1.

T ${\text{T}}_{2}^{*}$ values of fat and water and weighting bias correction factor			
**3 Tesla**			
	Fat ${\text{T}}_{2}^{*}$	Water ${\text{T}}_{2}^{*}$	${\text{T}}_{2}^{*}$ weighting bias correction factor (echo1/echo2/echo3/echo4/echo5)
V1	17.72	14.80	1.06/1.12/1.19/1.26/1.34
V2	17.83	14.13	1.09/1.19/1.30/1.42/1.55
V3	16.98	15.01	1.06/1.12/1.19/1.25/1.33
V4	12.01	11.77	1.04/1.08/1.12/1.17/1.21
V5	17.81	13.98	1.09/1.19/1.30/1.42/1.55
**7 Tesla**			
	Fat ${\text{T}}_{2}^{*}$	Water ${\text{T}}_{2}^{*}$	${\text{T}}_{2}^{*}$ weighting bias correction factor (echo1/echo2/echo3/echo4/echo5/echo6)
V6	7.53	8.88	0.93/0.86/0.80/0.75/0.69/0.64
V7	9.62	8.86	1.03/1.07/1.10/1.14/1.18/1.22
V8	9.12	8.58	1.02/1.04/1.07/1.09/1.12/1.14
V9	7.69	8.61	0.95/0.90/0.85/0.81/0.76/0.72
V10	8.79	8.86	0.99/0.98/0.98/0.97/0.96/0.95

## Data Availability

The data that support the findings of this study, including the ROIs, are openly available in “3T SMURF-based QSM data” and “7T SMURF-based QSM data” https://doi.org/10.7910/DVN/IEFCRA at and https://doi.org/10.7910/DVN/4HBJC6, references [78] and [79], respectively.
