# Supplementary material for: Quantitative susceptibility mapping of the head-and-neck using SMURF fat-water imaging with chemical shift and relaxation rate corrections
Source: Magn Reson Med. Author manuscript; Available in PMC 2022 Mar 1. (PMC7612304; doi:10.1002/mrm.29069)
Supplement: Supplementary Information [file EMS140943-supplement-Supplementary_Information.docx]

***Supporting Information Figure S1***


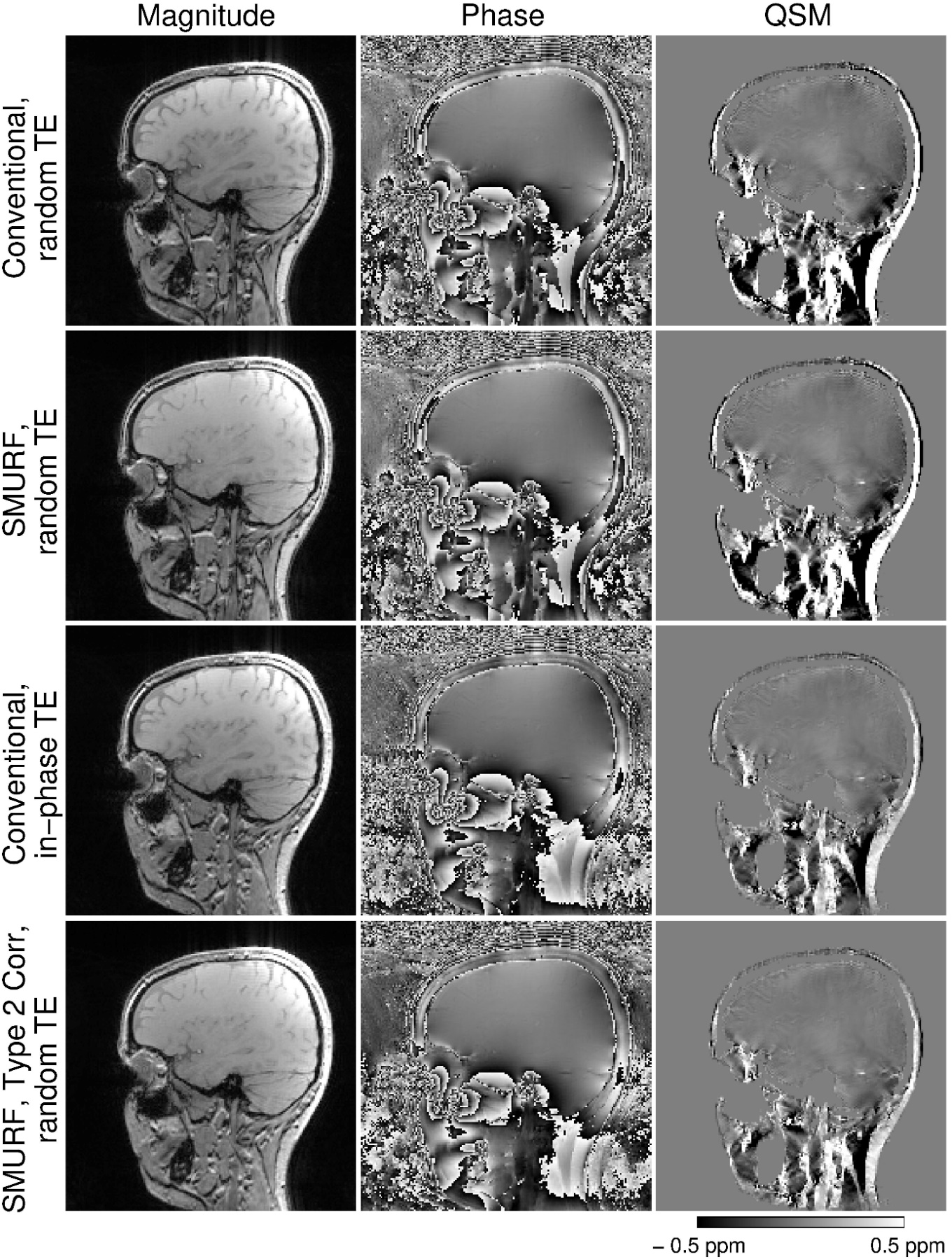


*Figure S1: Comparison of conventional GRE images (i.e. with broadband RF excitation) with recombined SMURF images at 3 Tesla. There is a very high degree of correspondence between the conventional image acquired at “random” echo-times (first row) and the recombined SMURF fat-water image acquired at the same, “random” echo-times (second row), demonstrating that the use of SMURF RF pulses and image reconstruction has no discernible effect on image contrast, signal phase or resulting susceptibility maps. Similarly, there was a high degree of correspondence between the conventional image acquired at the “in-phase” echo times (third row) and the “random” recombined SMURF fat-water image corrected for the Type 2 chemical shift artefact (fourth row), demonstrating that the correction effectively removed the phase discrepancy between water and fat.*

***Supporting Information Figure S2***


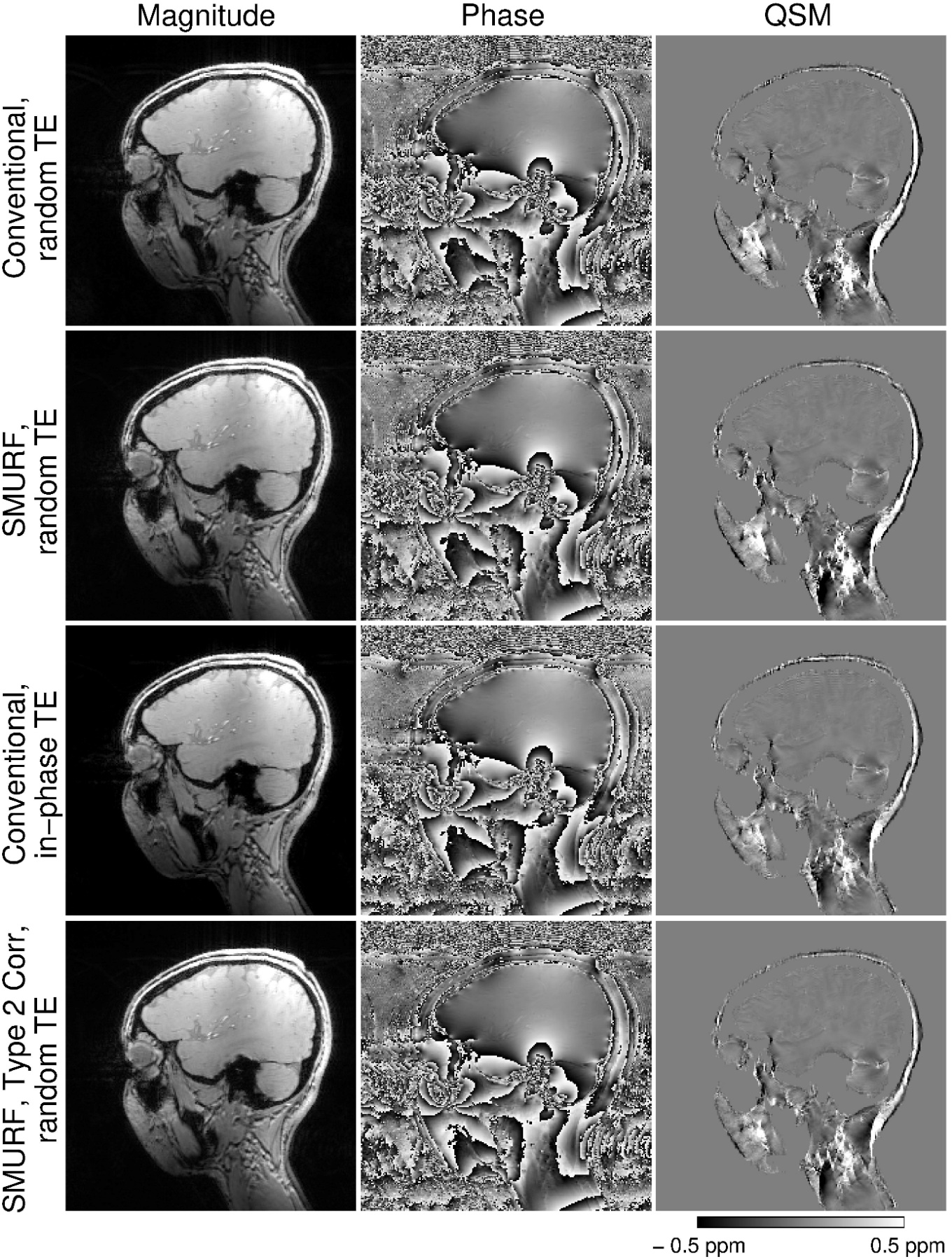


*Figure S2: Comparison of conventional GRE images (i.e. with broadband RF excitation) with recombined SMURF images at 7 Tesla. There is a very high degree of correspondence between the conventional image acquired at “random” echo-times (first row) and the recombined SMURF fat-water image acquired at the same, “random” echo-times (second row), demonstrating that the use of SMURF RF pulses and image reconstruction has no discernible effect on image contrast, signal phase or resulting susceptibility maps. Similarly, there was a high degree of correspondence between the conventional image acquired at the “in-phase” echo times (third row) and the “random” recombined SMURF fat-water image corrected for the Type 2 chemical shift artefact (fourth row), demonstrating that the correction effectively removed the phase discrepancy between water and fat.*

***Supporting Information Figure S3***


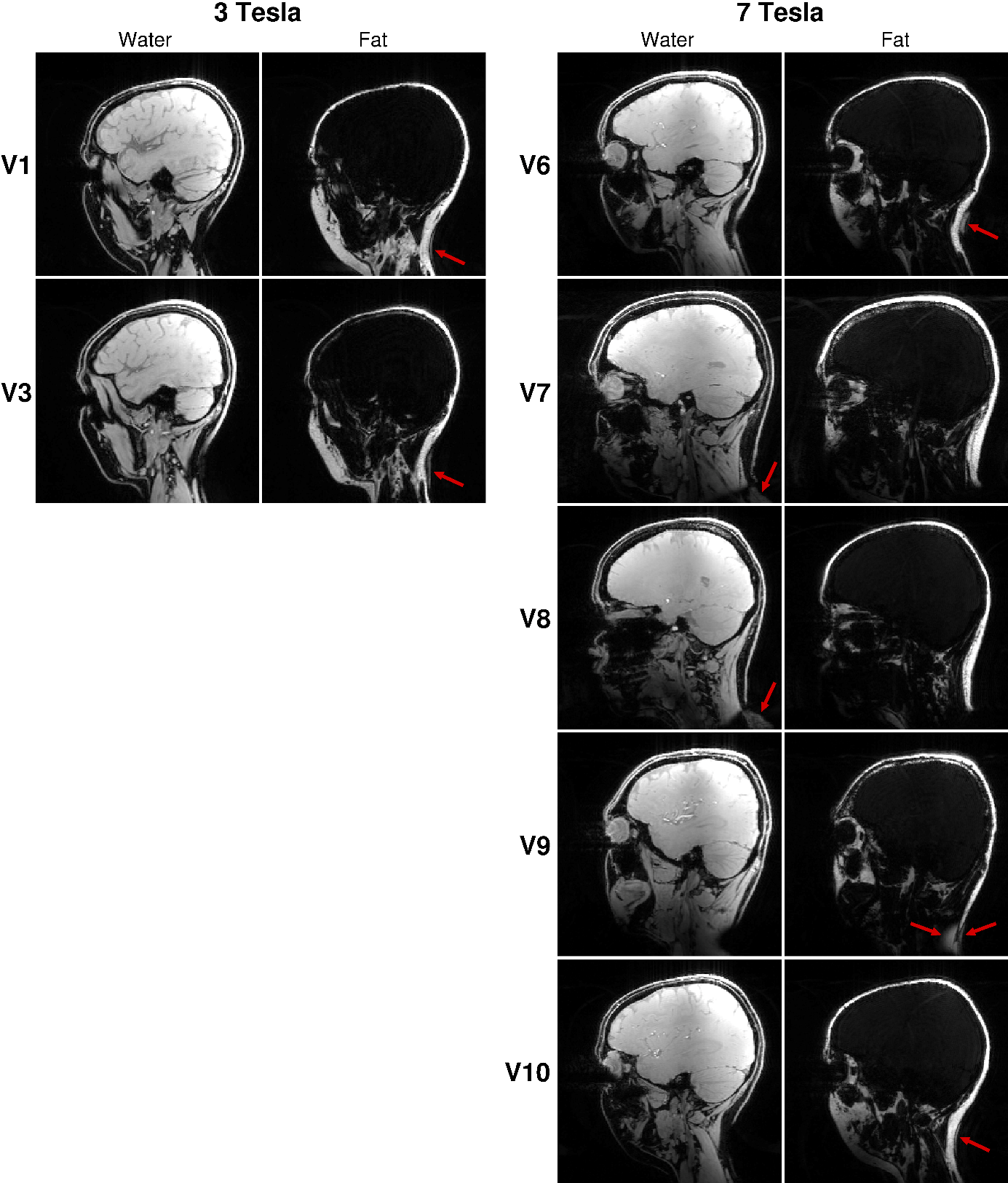


*Figure S3: Fat-water separation errors in the head-and-neck SMURF imaging. At 3 Tesla (left), in two out of five volunteers, fat and water signals are swapped in a small area of the very caudal, subcutaneous neck region. At 7 Tesla (right), in all five volunteers some local fat-water swaps are visible – either also in the very caudal, subcutaneous neck region (V6 and V10) or in the very inferior neck region. The swaps are, however, easy to identify and localized mostly outside the region of interest.*

***Supporting Information Figure S4***


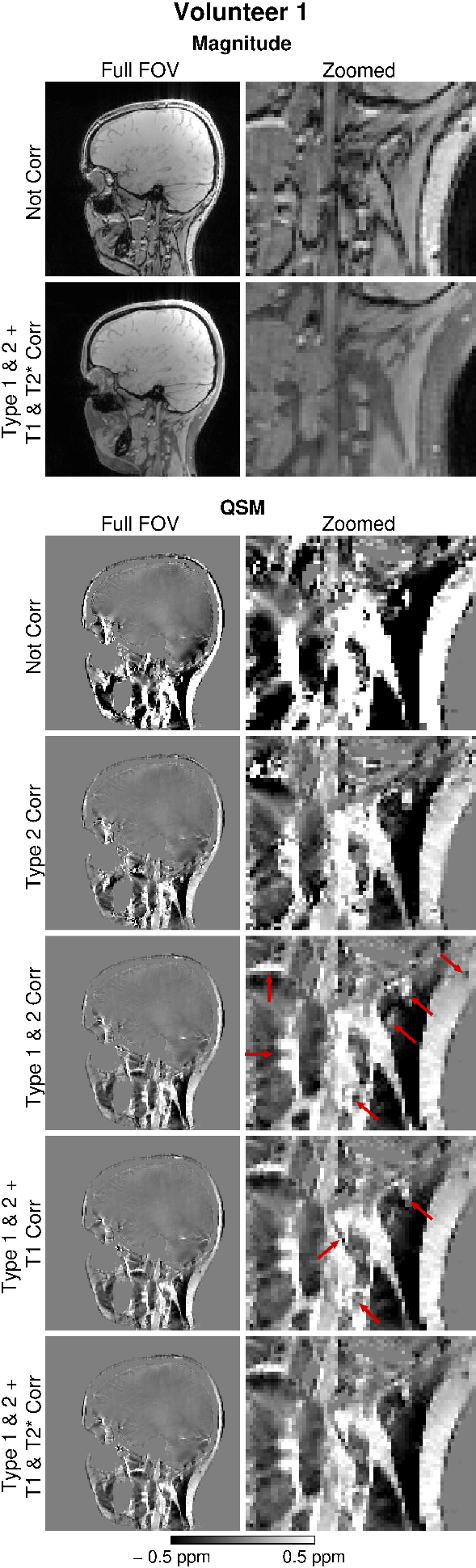


*Figure S4: Susceptibility maps of one 3T volunteer (V1) generated without any correction and with the individual corrections for chemical shift artefacts and relaxation rate bias applied. Without the corrections (row 3), the susceptibility maps are blurred and the values are much higher than when corrected for the Type 2 CSA (row 4). The susceptibility maps corrected for the Type 1 and Type 2 CSA clearly depict the paramagnetic fatty areas (row 5, red arrows). Corrections for the fat-water differences in T_1_ (row 6) and T_2_^*^ (row 7) relaxation rates remove the dominant influence of fat in the mixed voxels (row 6, red arrows). Note the small effect of the T_2_^*^ correction.*

***Supporting Information Figure S5***


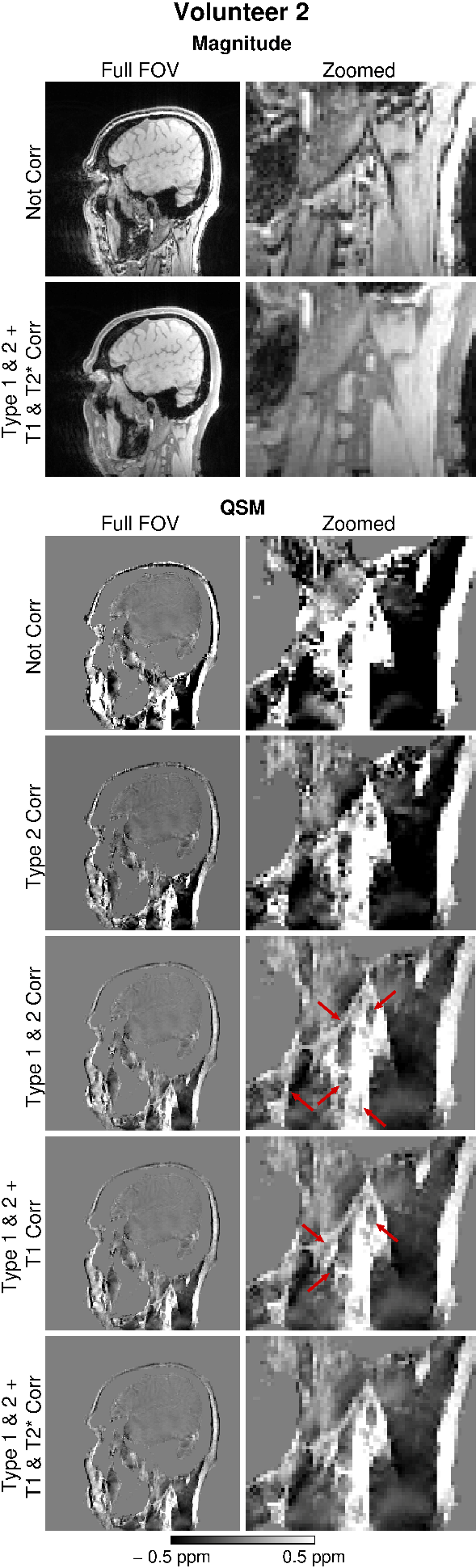


*Figure S5: Susceptibility maps of one 3T volunteer (V2) generated without any correction and with the individual corrections for chemical shift artefacts and relaxation rate bias applied. Without the corrections (row 3), the susceptibility maps are blurred and the values are much higher than when corrected for the Type 2 CSA (row 4). The susceptibility maps corrected for the Type 1 and Type 2 CSA clearly depict the paramagnetic fatty areas (row 5, red arrows). Corrections for the fat-water differences in T_1_ (row 6) and T_2_^*^ (row 7) relaxation rates remove the dominant influence of fat in the mixed voxels (row 6, red arrows). Note the small effect of the T_2_^*^ correction.*

***Supporting Information Figure S6***


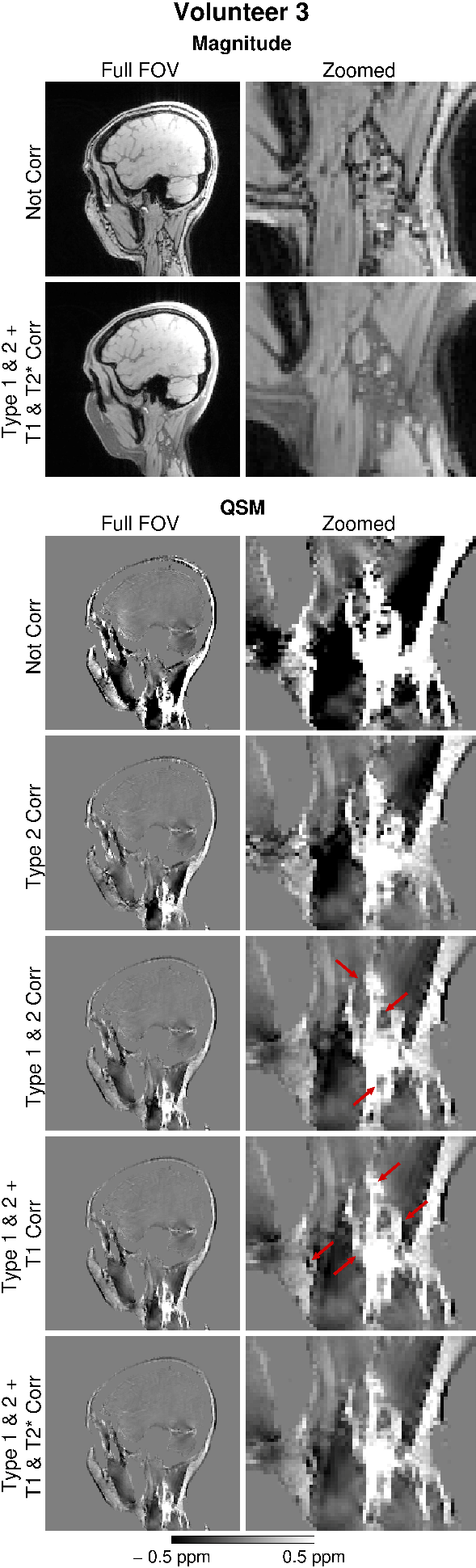


*Figure S6: Susceptibility maps of one 3T volunteer (V3) generated without any correction and with the individual corrections for chemical shift artefacts and relaxation rate bias applied. Without the corrections (row 3), the susceptibility maps are blurred and the values are much higher than when corrected for the Type 2 CSA (row 4). The susceptibility maps corrected for the Type 1 and Type 2 CSA clearly depict the paramagnetic fatty areas (row 5, red arrows). Corrections for the fat-water differences in T_1_ (row 6) and T_2_^*^ (row 7) relaxation rates remove the dominant influence of fat in the mixed voxels (row 6, red arrows). Note the small effect of the T_2_^*^ correction.*

***Supporting Information Figure S7***


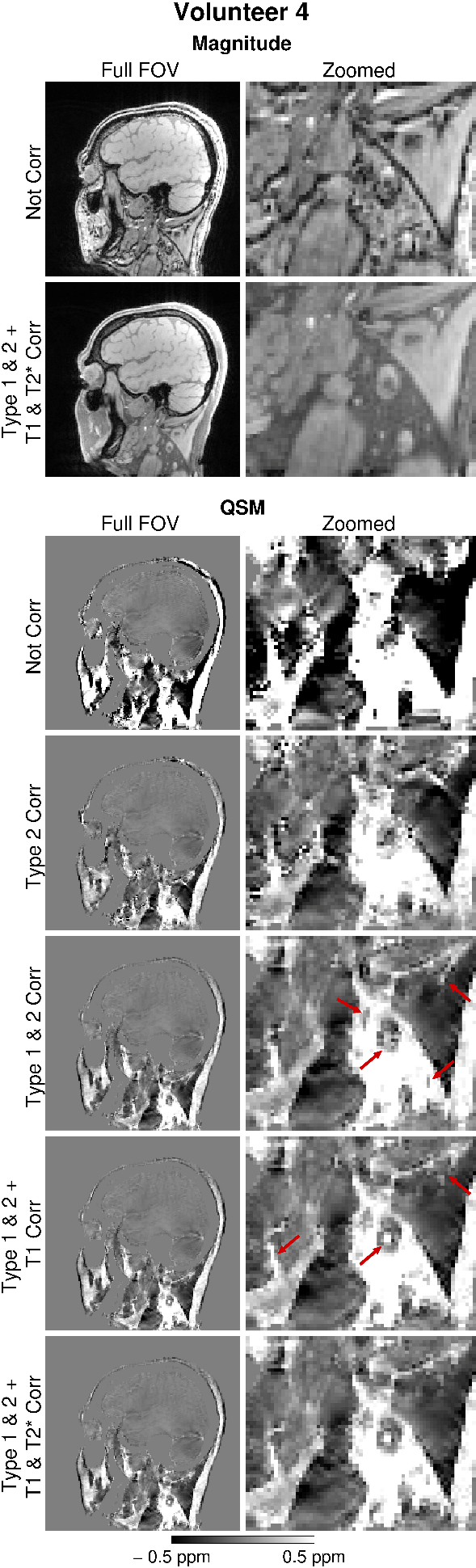


*Figure S7: Susceptibility maps of one 3T volunteer (V4) generated without any correction and with the individual corrections for chemical shift artefacts and relaxation rate bias applied. Without the corrections (row 3), the susceptibility maps are blurred and the values are much higher than when corrected for the Type 2 CSA (row 4). The susceptibility maps corrected for the Type 1 and Type 2 CSA clearly depict the paramagnetic fatty areas (row 5, red arrows). Corrections for the fat-water differences in T_1_ (row 6) and T_2_^*^ (row 7) relaxation rates remove the dominant influence of fat in the mixed voxels (row 6, red arrows). Note the small effect of the T_2_^*^ correction.*

***Supporting Information Figure S8***


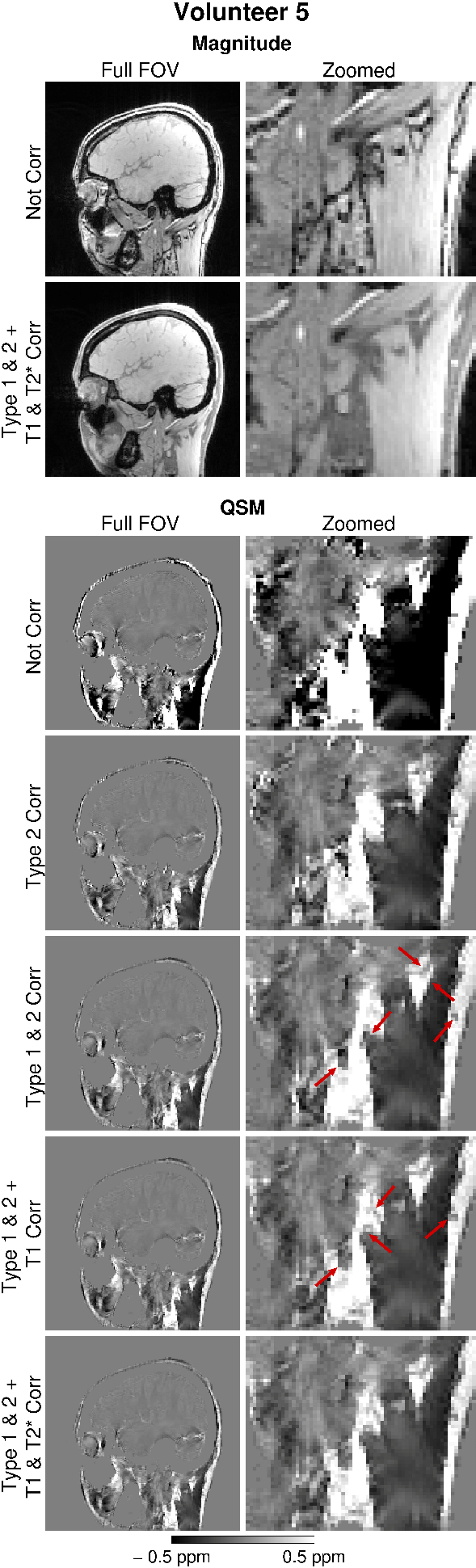


*Figure S8: Susceptibility maps of one 3T volunteer (V5) generated without any correction and with the individual corrections for chemical shift artefacts and relaxation rate bias applied. Without the corrections (row 3), the susceptibility maps are blurred and the values are much higher than when corrected for the Type 2 CSA (row 4). The susceptibility maps corrected for the Type 1 and Type 2 CSA clearly depict the paramagnetic fatty areas (row 5, red arrows). Corrections for the fat-water differences in T_1_ (row 6) and T_2_^*^ (row 7) relaxation rates remove the dominant influence of fat in the mixed voxels (row 6, red arrows). Note the small effect of the T_2_^*^ correction.*

***Supporting Information Figure S9***


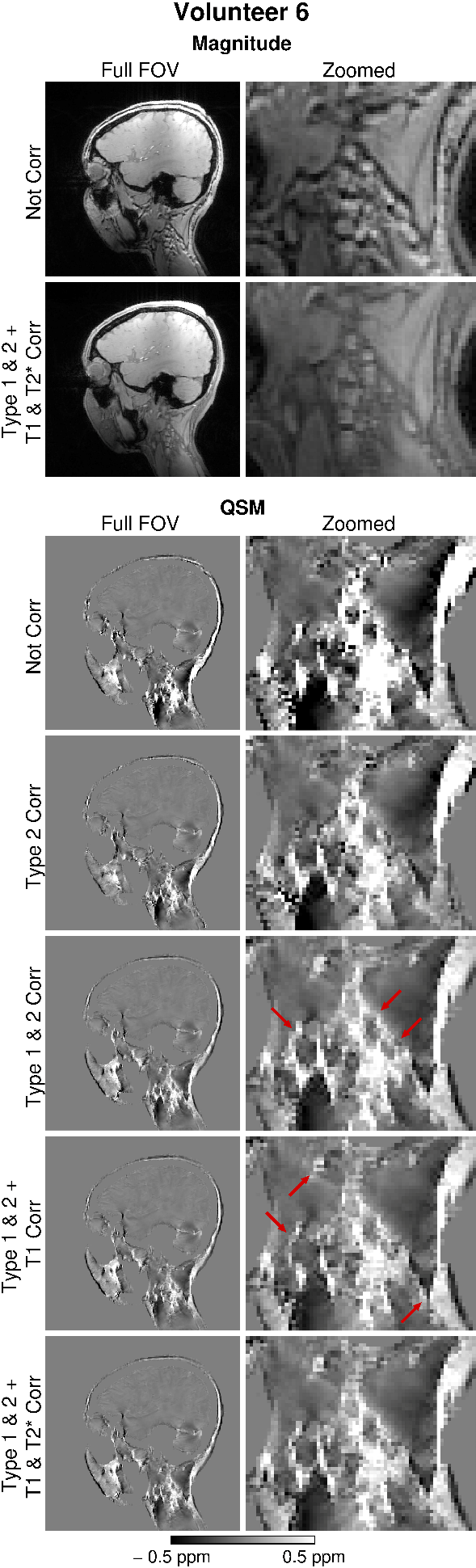


*Figure S9: Susceptibility maps of one 7T volunteer (V6) generated without any correction and with the individual corrections for chemical shift artefacts and relaxation rate bias applied. Without the corrections (row 3), the susceptibility maps are blurred and the values are much higher than when corrected for the Type 2 CSA (row 4). The susceptibility maps corrected for the Type 1 and Type 2 CSA clearly depict the paramagnetic fatty areas (row 5, red arrows). Corrections for the fat-water differences in T_1_ (row 6) and T_2_^*^ (row 7) relaxation rates remove the dominant influence of fat in the mixed voxels (row 6, red arrows). Note the small effect of the T_2_^*^ correction.*

***Supporting Information Figure S10***


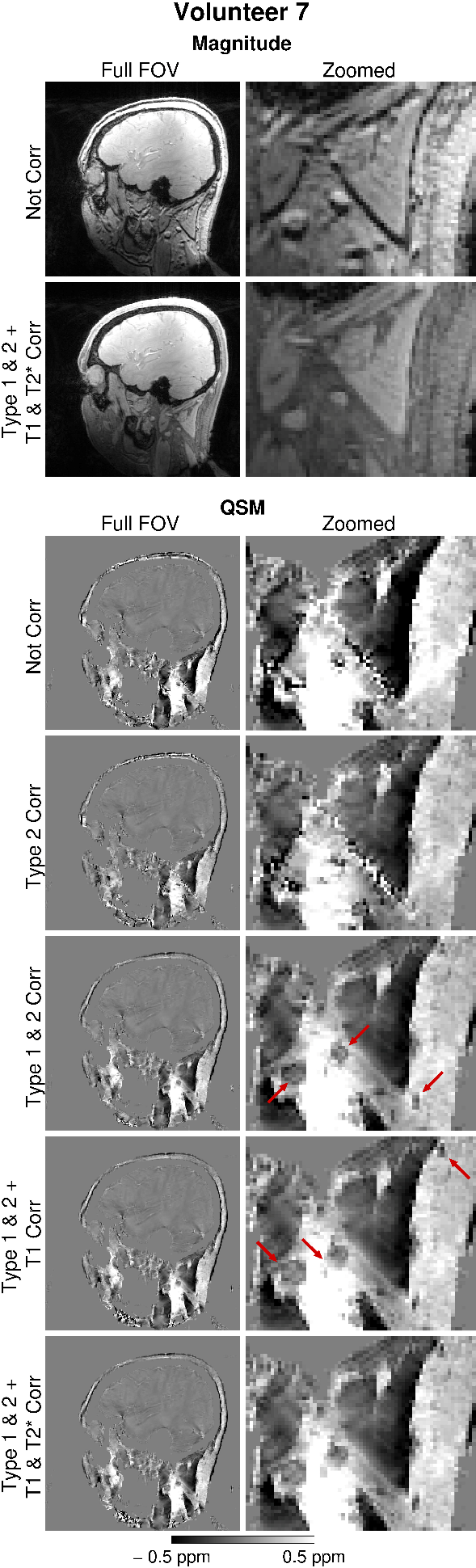


*Figure S10: Susceptibility maps of one 7T volunteer (V7) generated without any correction and with the individual corrections for chemical shift artefacts and relaxation rate bias applied. Without the corrections (row 3), the susceptibility maps are blurred and the values are much higher than when corrected for the Type 2 CSA (row 4). The susceptibility maps corrected for the Type 1 and Type 2 CSA clearly depict the paramagnetic fatty areas (row 5, red arrows). Corrections for the fat-water differences in T_1_ (row 6) and T_2_^*^ (row 7) relaxation rates remove the dominant influence of fat in the mixed voxels (row 6, red arrows). Note the small effect of the T_2_^*^ correction.*

***Supporting Information Figure S11***


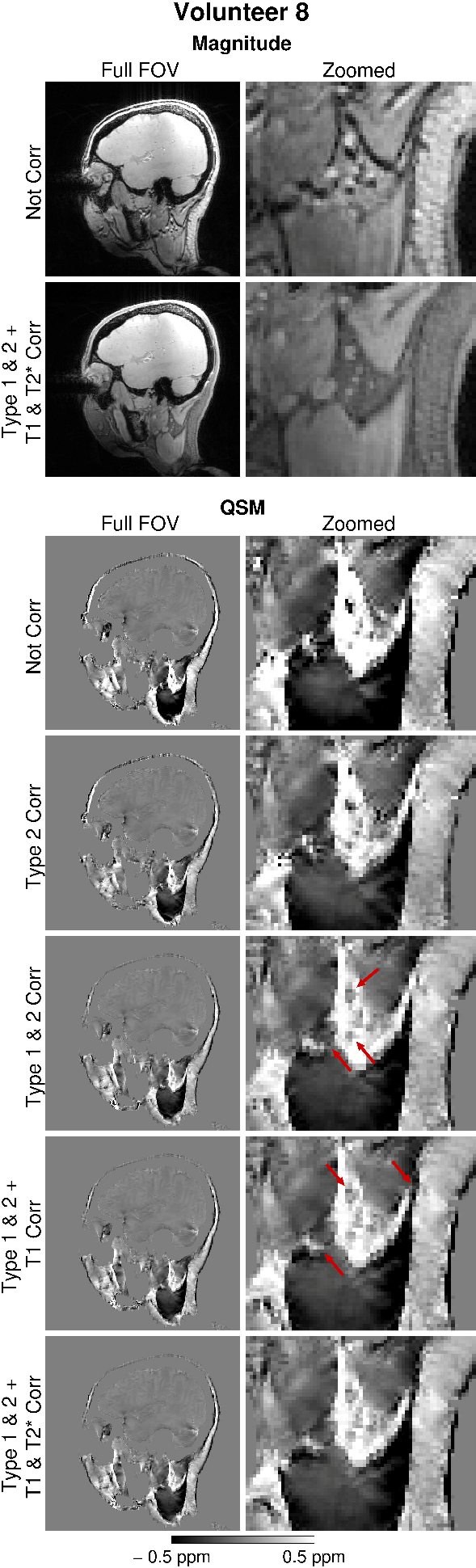


*Figure S11: Susceptibility maps of one 7T volunteer (V8) generated without any correction and with the individual corrections for chemical shift artefacts and relaxation rate bias applied. Without the corrections (row 3), the susceptibility maps are blurred and the values are much higher than when corrected for the Type 2 CSA (row 4). The susceptibility maps corrected for the Type 1 and Type 2 CSA clearly depict the paramagnetic fatty areas (row 5, red arrows). Corrections for the fat-water differences in T_1_ (row 6) and T_2_^*^ (row 7) relaxation rates remove the dominant influence of fat in the mixed voxels (row 6, red arrows). Note the small effect of the T_2_^*^ correction.*

***Supporting Information Figure S12***


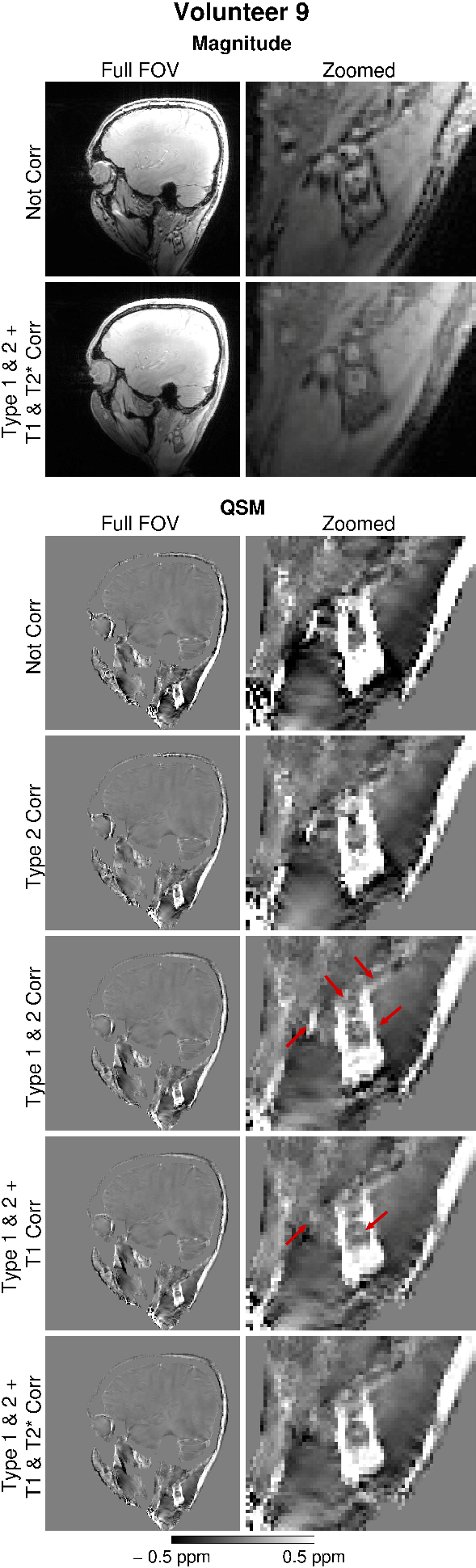


*Figure S12: Susceptibility maps of one 7T volunteer (V9) generated without any correction and with the individual corrections for chemical shift artefacts and relaxation rate bias applied. Without the corrections (row 3), the susceptibility maps are blurred and the values are much higher than when corrected for the Type 2 CSA (row 4). The susceptibility maps corrected for the Type 1 and Type 2 CSA clearly depict the paramagnetic fatty areas (row 5, red arrows). Corrections for the fat-water differences in T_1_ (row 6) and T_2_^*^ (row 7) relaxation rates remove the dominant influence of fat in the mixed voxels (row 6, red arrows). Note the small effect of the T_2_^*^ correction.*

***Supporting Information Figure S13***


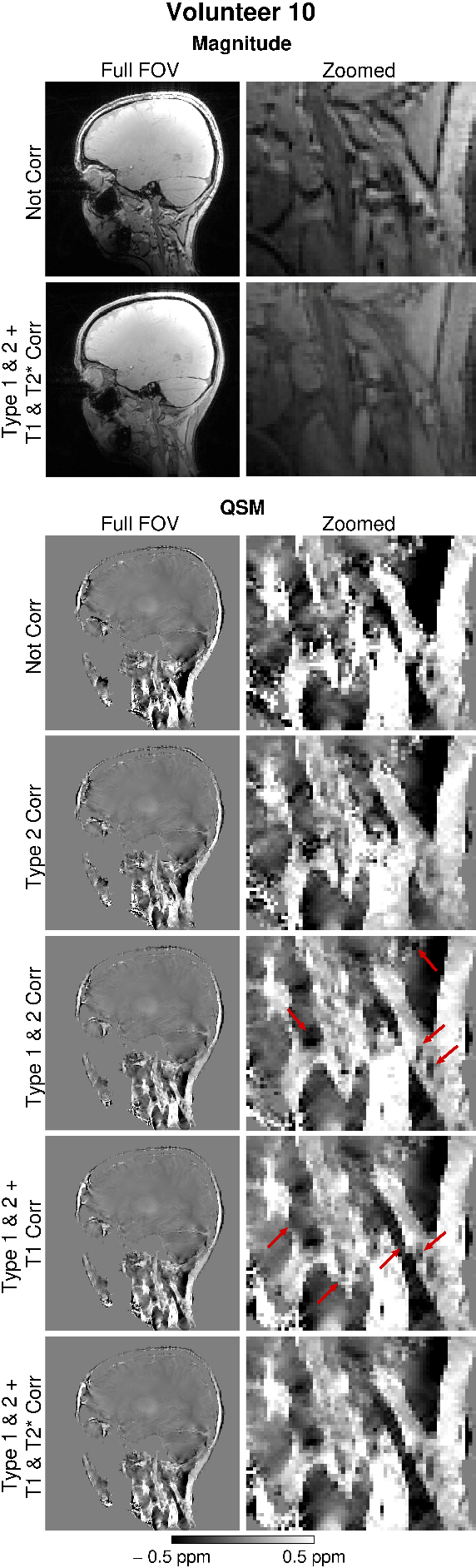


*Figure S13: Susceptibility maps of one 7T volunteer (V10) generated without any correction and with the individual corrections for chemical shift artefacts and relaxation rate bias applied. Without the corrections (row 3), the susceptibility maps are blurred and the values are much higher than when corrected for the Type 2 CSA (row 4). The susceptibility maps corrected for the Type 1 and Type 2 CSA clearly depict the paramagnetic fatty areas (row 5, red arrows). Corrections for the fat-water differences in T_1_ (row 6) and T_2_^*^ (row 7) relaxation rates remove the dominant influence of fat in the mixed voxels (row 6, red arrows). Note the small effect of the T_2_^*^ correction.*

***Supporting Information Figure S14***


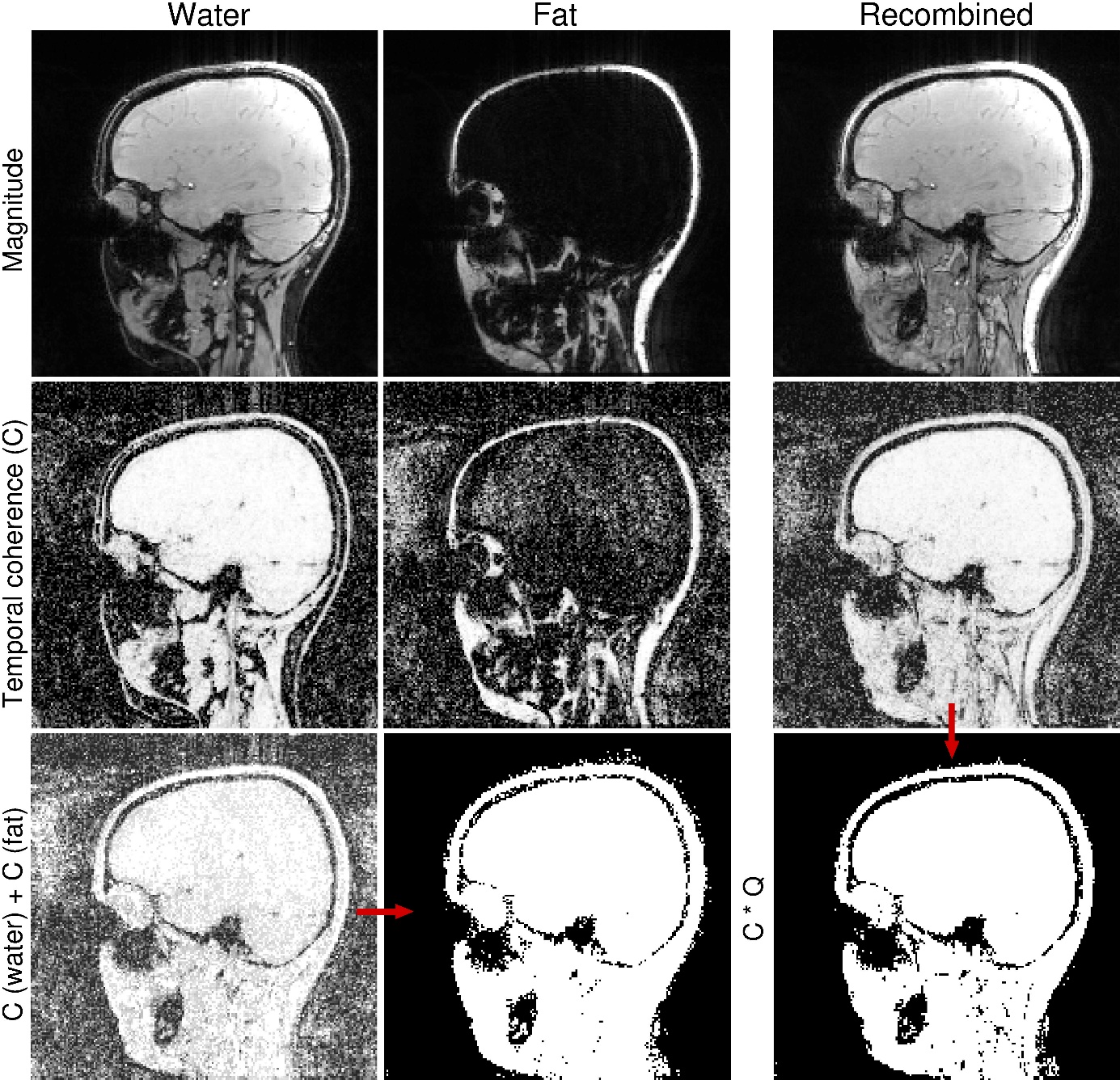


*Figure S14: The performance of the proposed masking approach when applied to the separated fat and water images (left two columns) and to the combined fat-water image (right column). The temporal coherence map of the recombined fat-water image shows low values in the mixed voxels at the fat-water borders, resulting in an exclusion of most of the mixed voxels of interest from the head-and-neck mask (right column, bottom). A joint temporal coherence map generated from the combination of separate fat and water temporal coherence maps shows high coherence values in mixed voxels, leading to these voxels being retained (middle column, bottom).*
